# Diversity of gut microbiomes in marine fishes is shaped by host‐related factors

**DOI:** 10.1111/mec.15699

**Published:** 2020-11-09

**Authors:** Qi Huang, Ronia C. Sham, Yu Deng, Yanping Mao, Chunxiao Wang, Tong Zhang, Kenneth M. Y. Leung

**Affiliations:** ^1^ School of Biological Sciences The University of Hong Kong Hong Kong China; ^2^ Department of Civil Engineering Environmental Microbiome Engineering and Biotechnology Lab The University of Hong Kong Hong Kong China; ^3^ College of Chemistry and Environmental Engineering Shenzhen University Shenzhen China; ^4^ State Key Laboratory of Marine Pollution and Department of Chemistry City University of Hong Kong Hong Kong China

**Keywords:** 16S rRNA gene, feeding habit, gut microbiome, host taxon, marine fishes, trophic level

## Abstract

Microorganisms in the gastrointestinal tract of animals play vital roles in food digestion, homeostasis and immune response regulation. Globally, there are 33,700 fish species, representing almost half of all vertebrate diversity and a wide range of physiologies, ecologies and life histories. To investigate gut microbiomes with high coverage, we performed 16S rRNA gene amplicon sequencing with 115 samples of 20 common marine fish species. The fish gut microbiome is a remarkably simple community with low microbial diversity (a maximum of 300 amplicon sequence variants only) and has up to 70% of unknown species in some fish species. The gut microbial community structure was significantly shaped by the combined influence of host‐associated factors, including the fish taxon (*p* < .001, *R^2^* = 0.16, *ω* ^2^ = 0.04), feeding habit (*p* < .001, *R^2^* = 0.06, *ω* ^2^ = 0.02) and trophic level (*p* < .01, *R^2^* = 0.04, *ω* ^2^ = 0.01), although the influence was subtle with a small effect size. The core gut microbiomes of different feeding habits were also previously discovered in animal‐associated and corresponding habitat samples. Certain energy metabolism pathways were enriched in herbivore/omnivore and zooplanktivore/zoobenthivore fishes, whereas lipid metabolism and glycan metabolism were enriched in zoobenthivore/piscivore fishes. Moreover, substantial taxonomic variability was found between the gut microbiomes of fish and animals, indicated by their low degree of shared microbiota. The data and observations reported herein pave the way for further investigations on the co‐evolution of fish gut microbiomes and their hosts, the physiological functions of gut microorganisms and the development of probiotics for improving the nutrition and health of aquaculture fish species.

## 1 INTRODUCTION

Multicellular higher organisms have coexisted with microbial communities on Earth for a long history of approximately 1.2 billion years (Butterfield et al., 1990). Interactions between multicellular organisms and microorganisms are expected to promote the generation of beneficial mutations. The external and internal body parts of multicellular organisms are large reservoirs of microbiota, especially the digestive tract, in which the microbial communities are more closely associated with the host than free‐living microbiota (Ley et al., 2008). Fishes serve as a large group of organisms that are indispensable to the exploration of the co‐evolution of hosts and their gut microbiomes in relation to the host's life history and feeding habits. According to FishBase (www.fishbase.org), there are approximately 33,700 fish species on Earth, accounting for over half of the total number of vertebrate species. The fish digestive tract houses many diverse microorganisms, including bacteria, archaea and fungi, which jointly create a complex microbial ecosystem (Wei et al., 2018). It has been reported that the population density of microbiomes in the marine fish gut is within the range of 10^7^–10^8^ cells per gram (Izvekova et al., 2007), suggesting that a vast hidden diversity of microbiomes resides in this habitat.

Microbial communities in fish guts can enhance host metabolic capacity through beneficial effects on nutrient digestion and assimilation, and can protect the host from invasive pathogens (Nayak, 2010). For instance, intestinal microbiomes can produce a variety of digestive enzymes that facilitate food digestion in fish, such as lipase, protease and cellulase (Ray et al., 2012). Germ‐free zebrafish were found to be unable to absorb protein macromolecules; however, they could restore their protein uptake capacity through the establishment of microbes, suggesting that gut microbiomes also contribute to host nutrient uptake and assimilation (Bates et al., 2006). The gastrointestinal tract is one of the major routes for the entry of some pathogens that may cause serious diseases such as vibriosis, enteric septicaemia and aeromoniasis (Nayak, 2010). In this regard, the gut microbiome plays a crucial role in the development and maturation of the gut immune system (gut‐associated lymphoid tissues), defending against pathogenic infection and inducing immunity (Rhee et al., 2004). Bates et al. (2007) found that zebrafish gut microbiomes also enhanced intestinal alkaline phosphatase expression, resulting in the dephosphorylation of bacterial lipopolysaccharides, thereby manipulating intestinal immune regulation to confront pathogenic infection. Cheesman et al. (2011) also noted that gut microbiomes facilitate epithelial cell proliferation by improving beta‐catenin stability.

The first report on fish gut microbiomes appeared in the 1920s and involved the study of the intestinal flora in haddock (Reed & Spence, 1929). During the 1950s–1990s, some studies focused on the determination of the composition of fish gut microbiomes and how they were influenced by diet, salinity and captive farming (Colwell, 1962; Margolis, 1953; Simidu & Hasuo, 1968). One of the pioneering studies in fish gut microbiota conducted by Yoshimizu and Kimura (1976) revealed that the intestinal microbiota in salmonids differentiated in different living environments and would undergo changes when their habitat changed in order to adapt fish themselves to the new habitat. Recently, by adopting advanced culture‐independent methods that have been recently developed, more detailed and comprehensive patterns of gut microbiomes have been uncovered, improving our knowledge about the complex gut microbiomes in fishes. For example, Zhang et al. (2018) revealed that the diversity of gut microbiomes in *Silurus meridionalis* significantly increased with host age, while Michl et al. (2017) reported that the gut microbial diversity of *Oncorhynchus mykiss* decreased with a reduction in nutrients.

To date, most studies on fish gut microbiomes have been carried out with a few targeted species of salmonids, including *Oncorhynchus mykiss* (Lyons et al., 2017; Michl et al., 2017), *Salvelinus alpinus* (Nyman et al., 2017) and *Salmo salar* (Llewellyn et al., 2016); laboratory model species, including *Gambusia affinis* (Carlson et al., 2017) and *Danio rerio* (Roeselers et al., 2011; Stephens et al., 2016); and wild‐caught species, including *Dicentrarchus labrax* (Gatesoupe et al., 2016) and *Siganus fuscescens* (Nielsen et al., 2017). Similar to the gut microbiomes of other types of animals (Sanders et al., 2015), the studied fish gut microbial communities were affected by a variety of factors, including the trophic level, feeding habit and taxon of the host fish (Dhanasiri et al., 2011; Egerton et al., 2018; Hovda et al., 2012; Schmidt et al., 2015; Sullam et al., 2012; Wong & Rawls, 2012; Yan et al., 2016). Given that fishes display very diverse taxonomic groupings with a wide range of feeding modes, a more comprehensive study of phylogenetically diversified marine fish species will enable us to reveal the robust correlation between the fish gut microbiome and host‐associated factors. The resulting collection of big data on the gut microbiome community associated with well‐defined sample metadata can help us to predict the feeding mode, taxon and even diet of a fish species based on its gut microbiome information.

A recent review on freshwater fishes suggested that the diversity of gut microbiomes is the highest in herbivorous fishes, followed by omnivorous and then carnivorous fishes (Wang et al., 2018). This is because herbivorous fishes require microorganisms such as *Clostridium*, *Citrobacter* and *Leptotrichia* to assist in the digestion of plant cellulose (Liu et al., 2016). In contrast, carnivorous fishes can easily digest and assimilate amino acids from a protein‐dominated diet without the need for these cellulose‐degrading bacteria. Nonetheless, the gut microbiomes of marine benthivorous and detritivorous fishes remain largely unknown. As benthivorous and detritivorous fishes live on the seabed and feed on benthic invertebrates and organic matter in sediment, respectively, it is hypothesized that their gut microbiomes are closely linked to the microorganisms occurring in the sediment and those associated with their diets, which consist of both animal and plant materials. Nonetheless, such a hypothesis has yet to be tested.

Here, this study revealed and compared the diversity of the gut microbial communities of 20 wild‐caught common marine fish species with 115 specimens collected from the marine environment of Hong Kong. 16S rRNA gene amplicon sequencing was utilized to identify the taxonomy of the gut microorganisms. Their patterns were elucidated with reference to the taxon, feeding habit and trophic level of these marine fish species to formulate generalizations regarding the relationship between gut microbiomes and their host fishes. The comparison of the predicted digestion‐related pathways among feeding habits and bacteria significantly associated with digestion‐related pathways was conducted to yield further insights into the relationships among fish feeding habits, gut microbiomes and digestion metabolism. The core microbiomes associated with each feeding habit and their common habitats were examined to gain insight into the potential sources of the gut microbiota. The similarities of the gut microbial communities in fish and other animals, especially their shared bacteria, were also addressed. The results allow us to comprehensively examine the robust correlations between gut microbiomes and the taxon/feeding habit/trophic level of the host. This study greatly expands the current database of fish gut microbiome information and sheds new light onto the hidden diversity of marine fish gut microbiomes, which can facilitate future studies on the manipulation of beneficial microorganisms with the aim of influencing the precise use of probiotics in fish farming practices.

## 2 MATERIALS AND METHODS

### 2.1 Fish sampling and gut content collection

In this study, a total of 115 individuals of fishes belonging to 20 wild‐caught marine fish species were sampled from the coastal waters of Hong Kong. The samples were either collected by a research trawler (Tao et al., 2018) or by local fishermen using gillnets, purse seine netting or longlines. The research‐related trawling work was permitted by the Agriculture, Fisheries and Conservation Department. Most of the fishes were dead on board and stored at −20°C in an ice box before being transported to the laboratory. The fishes were sorted and identified according to their morphological characteristics. These 115 fish samples belonged to 5 fish orders (Aulopiformes, Clupeiformes, Scorpaeniformes, Perciformes and Pleuronectiformes) and exhibited varied feeding habits (herbivore/omnivore, zooplanktivore/zoobenthivore, zoobenthivore, zoobenthivore/piscivore and piscivore) and different trophic levels (Table S1).

The fishes were processed in a timely manner in order to reduce the change in composition of gut microbial communities over time and with perturbation (Clements et al., 2014). Before dissection, 70% ethanol was applied to the body surface of the fish samples. The fishes were then dissected with individual‐use insect pins or individual‐use scalpels and forceps depending on the fish size. The digestive tract from the stomach to the hindgut was removed intact. The attached organs, such as the liver, were carefully removed. The gastrointestinal contents were squeezed out from the digestive tract and then washed in 70% ethanol and sterile water to disinfect the samples from transient bacteria before being stored at −20°C or directly undergoing DNA extraction. Dorsal muscle tissues were also collected for stable isotope analysis (SIA). The average time from extracting the contents to freezing was about 15 min (ranging from 10–20 min) per fish.

### 2.2 Stable isotope analysis

The SIA was conducted using the stable isotope ratio mass spectrometry (SIRMS) facility at the School of Biological Sciences of the University of Hong Kong to determine the trophic level of the fish species. Dorsal muscle samples were taken from each fish specimen and dried using a freeze drier, and the dried tissues were then ground. Approximately 1.00 mg (±0.10 mg) of dried sample was weighed and subjected to SIA with SIRMS (EuroVector, model EA3028). δ^15^N is enriched in a stepwise manner from prey to consumers, allowing the determination of trophic levels, while δ^13^C may differ among food resources and show little enrichment during trophic transfer, allowing the tracing of carbon and energy sources (Perkins et al., 2018). The methods for the determination of the δ^13^C and δ^15^N content in the samples followed those described by Perkins et al. (2018).

### 2.3 DNA extraction and 16S rRNA gene amplicon sequencing

DNA from the gut content samples was extracted with the FastDNA^®^ SPIN Kit for Soil (MP Biomedicals™, USA) following the protocol described by the manufacturer, as our previous study showed that the performance of this kit was better than that of other commercially available kits in terms of the recovery and purity of microbial DNA (Guo & Zhang, 2013). DNA concentration and quality were measured with a Qubit fluorometer and NanoDrop spectrophotometer, respectively. The V3‐V4 hypervariable region of the 16S rRNA of the qualified DNA samples was amplified with the primers 341F (5’‐CCTACGGGNGGCWGCAG‐3’) and 805R (5’‐GACTACHVGGGTATCTAATCC‐3’) and then sequenced using the Illumina MiSeq PE 300 platform with 30,000 raw reads per sample. Furthermore, two negative control samples were included to check the background and processing contamination during DNA extraction and PCR amplification. Details about the negative controls and other quality control treatments are presented in Appendix S1.

### 2.4 Bioinformatic and statistical analysis

The raw sequences were analysed with the QIIME 2 pipeline (https://qiime2.org/) (Caporaso et al., 2010). As a quality control procedure, low‐quality bases, chimeras and ambiguities were removed before the data were analysed. A feature table of amplicon sequence variants (ASVs) was constructed with the DADA2 pipeline (Callahan et al., 2016), the features were annotated with the Greengenes 13_8 database (DeSantis et al., 2006) using the RDP classifier (Wang et al., 2007), and then, mitochondria and chloroplast sequences were removed.

The Kruskal–Wallis test and Dunn's test were used to evaluate the differences in diversity among categories (fish taxon, feeding habit and trophic level) in IBM SPSS Statistics for Windows, version 22 (IBM Corp, 2013). Canonical correspondence analysis (CCA) were conducted by function “capscale.gen” in “vegan” package with squared‐root transformed Bray–Curtis dissimilarity in R. Spearman's correlation analysis was conducted by “cor.test” function in R. The ASVs representing the different fish species/feeding habits/trophic levels were identified with LEfSe (Segata et al., 2011) and the “labdsv” package in R.

Permutational multivariate analysis of variance (PERMANOVA) based on Bray–Curtis similarity matrix was conducted to test the importance of fish taxon, feeding habit and trophic level as factors to influence the fish gut microbiomes using PRIMER‐E PERMANOVA + software (Anderson et al., 2008). As the sample size is unequal across species, type III sum of squares (SS) were adopted with 999 permutations. The significance was determined by a Monte Carlo test. To qualify the effect size for each factor, both coefficients of determination *R* ^2^ and omega‐squared *ω* ^2^ were calculated (Kelly et al., 2015; Zhang & Alekseyenko, 2017). The homogeneity of multivariate dispersions for each factor was tested by the “PERMDISP” tool in PRIMER‐E.

The functional profiles of the microbial communities based on 16S rRNA gene sequences were predicted using the latest version of PICRUSt2 (v2.3.0‐b) according to the KEGG Orthology (Caicedo et al., 2020). Heatmaps of KEGG level 1 and level 2 pathways and the digestion‐related genes were plotted using the “pheatmap” package in R. The digestion‐related genes were selected based on a study of the gut microbiomes of the blunt snout bream (Wei et al., 2018). Kruskal–Wallis tests and Dunn's tests were also used to test the relative abundance of digestion‐related gene functions to identify differences among the various feeding habits. Further, the significantly higher digestion‐related functional pathways determined by the Kruskal‐Wallis and Dunn's tests were considered as the enriched pathways. The statistically significant associations between relative abundance of digestion‐related functions and the ASV were identified by HAIIA (Rahnavard et al.).

To compare the fish and other animals’ gut microbiomes, a total of 717 16S rRNA samples covering gut microbiome data for fish, bats, bears, birds, cattle, humans, lizards and monkeys were included in this study, which were downloaded as Earth Microbiome Project (EMP) Greengenes‐closed‐referenced biom files (ftp://ftp.microbio.me/emp/release1/otu_tables/closed_ref_greengenes/) (Thompson et al., 2017). To combine the data from the 115 fish samples collected in this study with the downloaded data sets, closed‐reference clustering against Greengenes version 13.8 with 97% similarity was applied in QIIME 2 for both EMP data and fish gut microbiome data in this study. The “make_otu_network.py” script in QIIME 1 was used to generate the ASV network files, and then, the generated files were passed into Cytoscape (https://cytoscape.org/) (Shannon et al., 2003) to analyse and display how the ASVs were partitioned among animals. The intersecting ASV distribution among fish and other animals was visualized in UpSetR (Conway et al., 2017).

The core microbiomes associated with each feeding habit were determined by both their highest abundance and highest prevalence within the corresponding feeding habits. To better understand the common lifestyles of these core gut bacteria, the habitats were determined based on the three closest BLASTn hits against the NCBI NT database for each core microbiome ASV. Using this method, a total of 35 core ASVs and 87 closest BLASTn hits were characterized.

## 3 RESULTS

### 3.1 Taxonomic composition of fish gut microbiomes

The percentage of taxonomically unassigned ASV sequences generally increased with decreasing host taxonomic resolution from the phylum to species level (Figure S1). The ASVs were well annotated above the order level, at which fewer than 20% sequences were unidentified bacterial genomes. There was also a large percentage (over 70%) of unassigned ASVs at the genus level, especially for three fish species, that included *Siganus canaliculatus*, *Solea ovata* and *Parachaeturichthys polynema*.

**FIGURE 1:**
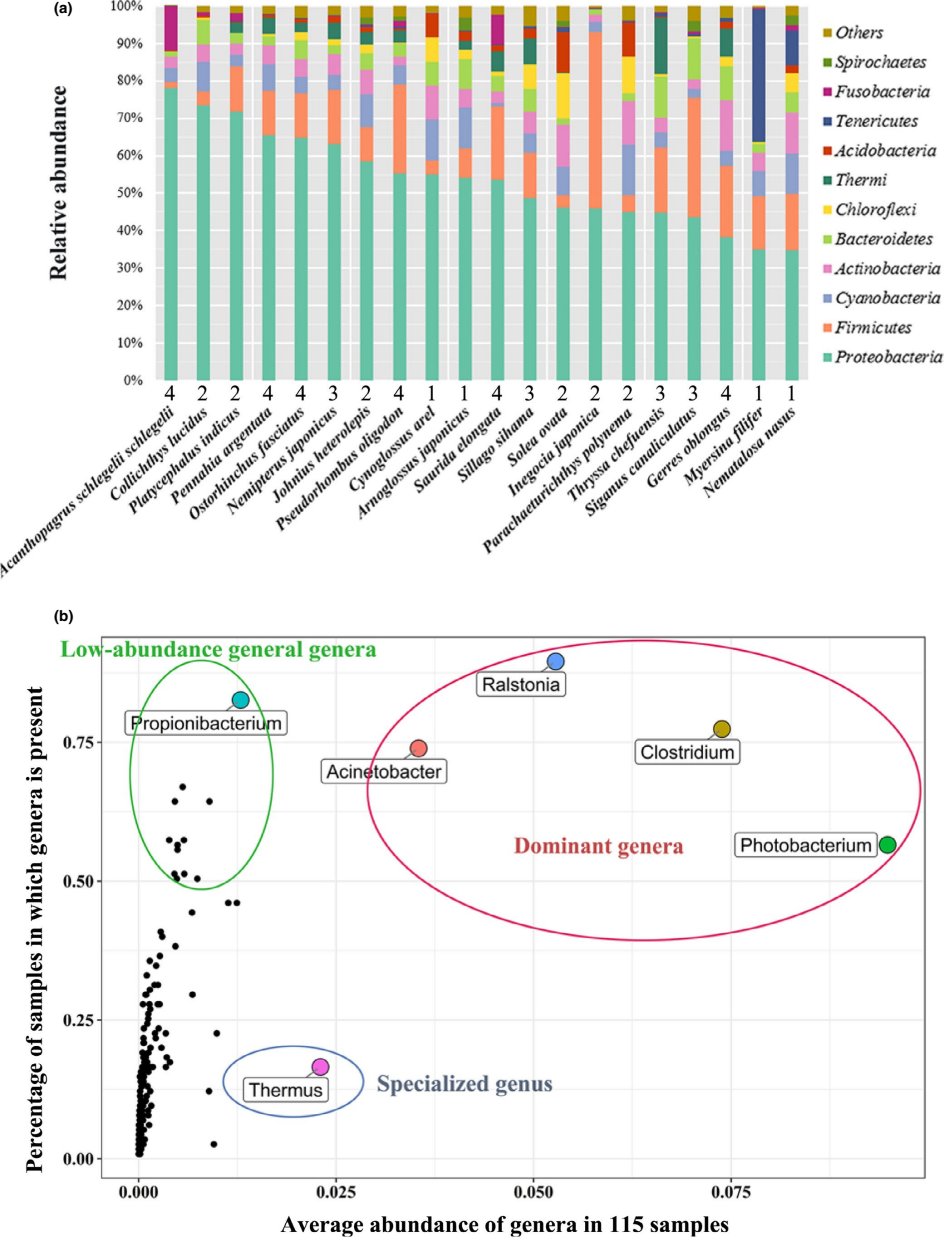
General profile of the gut microbial community of 20 fish species. (a) Relative abundance of gut microbiomes at the phylum level in 20 marine fish species. Proteobacteria and Firmicutes are the dominant phyla in the fish gut microbiomes. Numbers labelled at the x‐axis indicate the trophic level from level 1 consumer to level 4 consumer. (b) Abundance against the prevalence of bacteria at the genus level in the fish guts among the 115 samples. Dominant genera: both high abundance and high prevalence; Low‐abundance general genera: low abundance and high prevalence; Specialized genus: high abundance and low prevalence [Colour figure can be viewed at wileyonlinelibrary.com]

The bacterial richness in the gut represented by the cumulative ASVs revealed moderately diverse gut communities among the 20 sampled marine fish species (Figure S2). The maximum total number of ASVs in the fish gut samples was approximately 300, indicating a community composition with low diversity when compared with that in other types of animals such as humans (1,000–2,000 95% OTUs) (De Filippo et al., 2010), chickens (~900 97% OTUs) (Pan & Yu, 2014), turkeys (~500 97% OTUs) (Pan & Yu, 2014) and environments such as activated sludge (~1,000 97% OTUs) (Zhang et al., 2012). Eleven phyla were observed in the core gut microbiota of all 20 fish species, accounting for over 90% of the total number of annotated sequences (Figure 1a). Despite certain shared features, for example, Proteobacteria (average 52%) and Firmicutes (average 16%) being the dominant (most abundant and prevalent) phyla across fish species, the gut microbial communities of the different fish species showed a high degree of taxonomic variability. For instance, the filamentous shrimpgoby (*Myersina filifer*) harboured a considerable proportion of Tenericutes (average 35%), a phylum of gram‐negative, obligate cell‐associated bacteria that have lost their cell walls and many biochemical pathways, becoming highly dependent on their host cells, indicating a classical host–microbiome association.

Among the fraction of ASVs annotated at the genus level (average 56%) (Figure S2), six genera distinguished themselves from the others based on the relationship between the relative abundance and prevalence percentage (Figure 1b). Four genera, *Clostridium*, *Photobacterium*, *Ralstonia* and *Acinetobacter*, were the dominant genera, as they had both a high abundance (average >2.5% of sequences) and high prevalence percentage (existing in >50% of samples), while *Propionibacterium* was a representative low‐abundance general genus. An interesting finding was that the genus *Thermus* comprised approximately 13.4% of the total gut bacteria in the marine fish *Thryssa chefuensis*, which is distinguishingly higher than that in other fishes.

When considering the ASVs, the four most abundant ASVs were classified as members of the genus *Ralstonia* and the cyanobacterial lineage ML635J‐21, being commonly found in over 47% of all specimens (Figure S3). According to the BLASTn results against the NCBI NT database, the seven best hits with 99.776% identity and 100% (446 bp) alignment length suggested that the ML635J‐21‐clade ASV came from common bacteria that are also found on the surface of corals, deep‐sea polymetallic nodules, and volcanic rock and in deep‐well radioactive waste disposal sites without detailed taxonomic annotation, indicating the widespread distribution and novelty of this bacterial genome. Future studies to identify its complete genome and characterize its functions in the fish gut and the environment would be worthwhile and intriguing.

### 3.2 The effect of fish taxon on gut microbial communities

**FIGURE 2:**
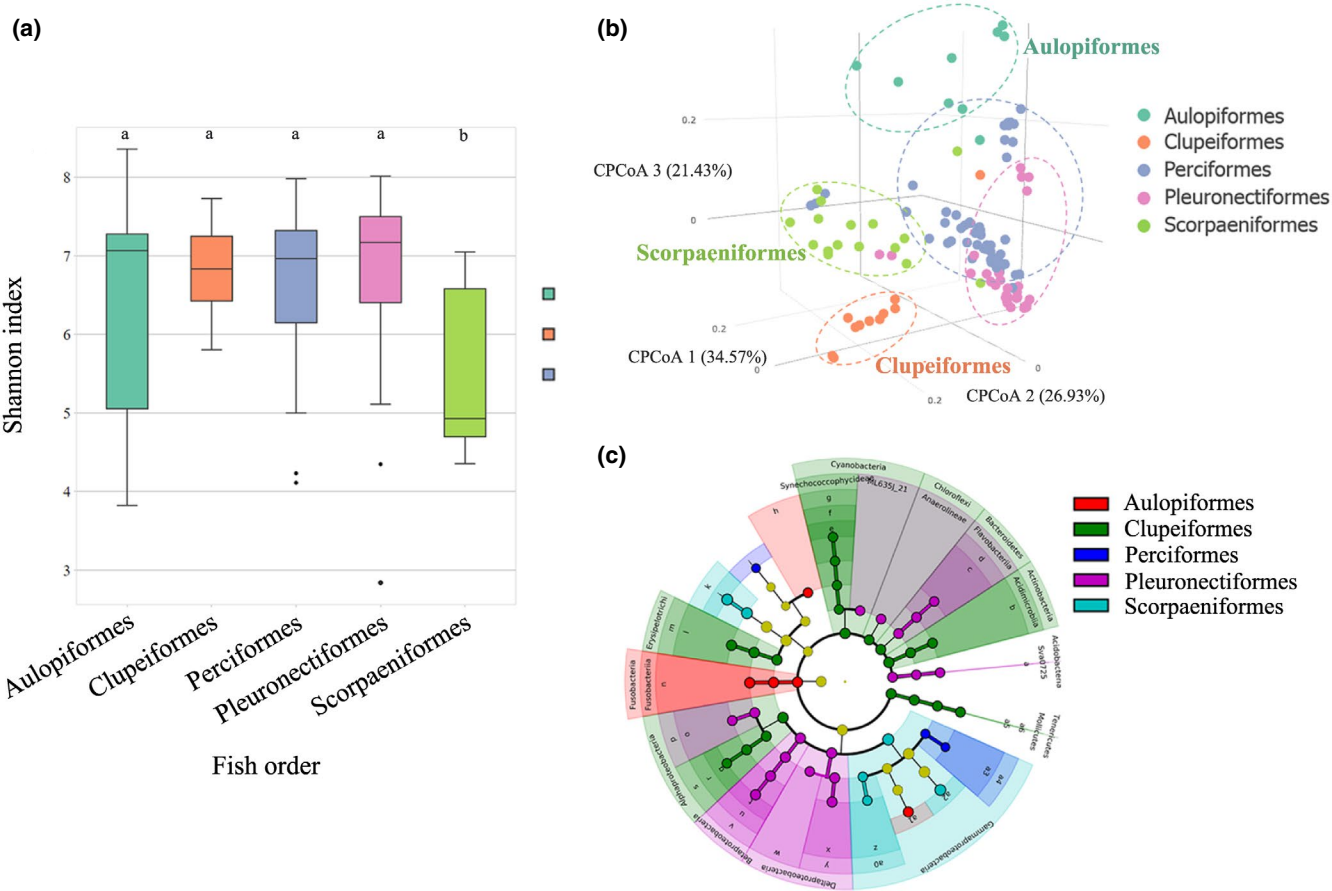
The pattern of fish gut microbiomes shaped by host taxon. (a) Box plots of the Shannon index of the gut microbiomes in the tested fishes among five orders. Kruskal–Wallis test and Dunn's test (*p* < .05). The letters “a” and “b” represent Dunn's test grouping results. (b) Clustering pattern among all fish gut microbiome samples categorized by five fish orders. Canonical correlation analysis was used, and distance was based on Bray–Curtis distance. (c) Bacterial clades most likely to explain the differences among fish orders. LDA (linear discriminant analysis) was used using LefSe (linear discriminant analysis effect size), and ASV with LDA score (log 10) larger than 3.4 was identified as the discriminative ASV [Colour figure can be viewed at wileyonlinelibrary.com]

According to the PERMANOVA results, fish species identity significantly explained the differences in the overall bacterial community composition in the fish gut based on Bray–Curtis distance matrixes (*p* < .001, *R^2^* = 0.16, *ω* ^2^ = 0.04) (Table S2). When the ASVs were compared at the species level, high levels of both interspecific and intraspecific variability in the microbial composition were observed (Figure S4). At the fish order level, Scorpaeniformes hosted the least diverse communities, while no significant difference in diversity was found among the four other fish orders (Figure 2a, Kruskal–Wallis test and Dunn's test, *p* < .05). Since Scorpaeniformes mainly includes piscivorous fish at a relatively high trophic level, the main metabolic differences related to the lack of consumption of vegetation might explain this trend.

Given the homogeneity of multivariate dispersion of fish order was rejected (*p* < .05), canonical correlation analysis (CCA) was used to help evaluate whether the gut microbial communities were influenced by the difference in composition between orders or within orders. The three orders Aulopiformes, Clupeiformes and Scorpaeniformes were well separated, indicating that their gut microbiomes had distinguishing composition and structure patterns related to the taxonomic lineage of their hosts (Figure 2b, Bray–Curtis distance; PERMANOVA, *p* < .001; PERMDISP, *p* < .05). To further identify the critical bacteria differentiating the fish orders, discriminative bacterial clades among the fish orders (Figure S5) were identified using a nonparametric test of significance and linear discriminant analysis implemented in LEfSe with a stricter cut‐off than the default (LDA score (log 10)> 3.4) (Figure 2c). The analyses identified 108 discriminatory bacterial clades, of which 14 (13%), 52 (48%) and 7 (6%) distinguished Aulopiformes, Clupeiformes and Scorpaeniformes, respectively, from all other fish orders. The discriminative clades for Clupeiformes largely belonged to Proteobacteria, Actinobacteria and Bacteroidetes despite the highest abundance of Proteobacteria in the entire data set. Aulopiformes was distinguished by *Lactococcus*, *Siphonobacter*, *Fusobacteriales* and *Methylacidiphilales*, while Scorpaeniformes contained more *Clostridium*, *Photobacterium*, *Francisellaceae* and *Vibrio*.

### 3.3 Feeding habit drives microbial taxonomic clustering

**FIGURE 3:**
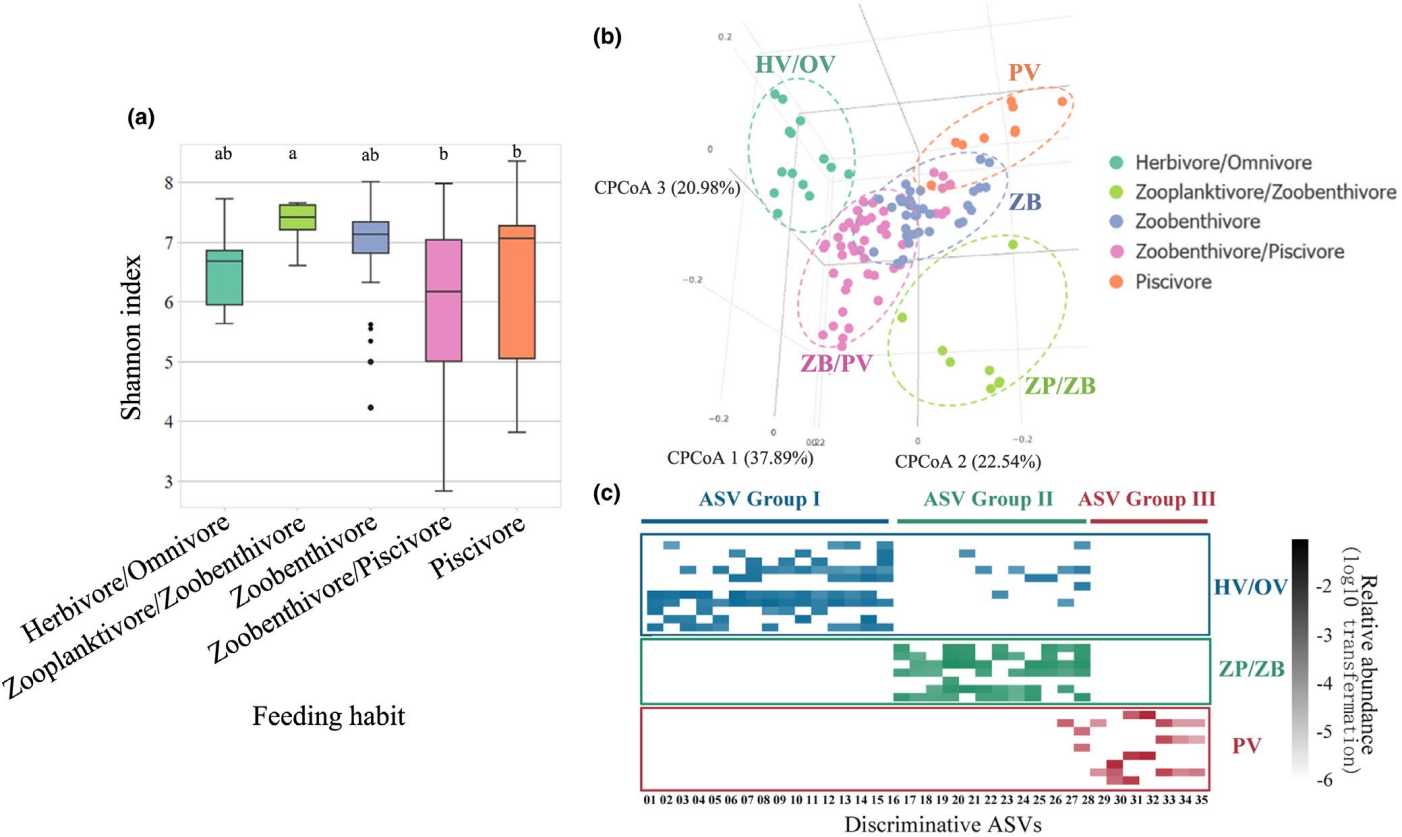
The pattern of fish gut microbiomes shaped by feeding habit. (a) Box plots of Shannon index of the gut microbiomes in fishes among five different feeding habits. Kruskal‐Wallis test and Dunn's test (*p* < .05). The letters “a” and “b” represent Dunn's test grouping results. (b) Clustering pattern among all fish gut microbiome samples categorized according to the five feeding habits. Canonical correlation analysis was used, and distance was based on Bray‐Curtis distance. (c) Abundance heatmap of discriminative gut microbiomes detected among the three distinguishing fish feeding habits (*p*‐value < 0.01) [Colour figure can be viewed at wileyonlinelibrary.com]

Zoobenthivores/piscivores hosted a wide range of bacterial diversity (Figure 3a), and zooplanktivores/zoobenthivores showed the highest bacterial diversity on average. Zoobenthivores/piscivores and piscivores shared comparable diversity, which was slightly lower than that of zooplanktivores/zoobenthivores (*p* < .05). This observation again conforms to the pattern that carnivore gut microbiomes are less diverse than herbivore gut microbiomes (Ley et al., 2008).

The gut microbial communities associated with the different feeding habits, especially the herbivore/omnivore, zooplanktivore/zoobenthivore and piscivore feeding habits, were significantly separated at the ASV level (Figure 3b, Bray–Curtis distance; PERMANOVA, *p* < .001; PERMDISP, *p* > .05), suggesting that they have dissimilar microbiome composition and structure and the occurrence of a strong nutritional–environmental effect. Along CCA axis 1, a dietary gradient was observed: distance typically increased from herbivores/omnivores, zooplanktivores/zoobenthivores, zoobenthivores and piscivores to zoobenthivores/piscivores. This trend alone explained most of the taxonomic diversity among the gut microbial communities (37.89% of the total variance along CCA axis 1) and significantly discriminated the herbivores/omnivores from the other feeding habit groups.

In addition to the ordination of fish gut microbiomes by different feeding habits, ASVs that can discriminate individual feeding habits were identified with a more robust statistical method (Figure 3c). Thirty‐five ASVs were selected as discriminative indicators of three feeding habits, which was consistent with the dissimilarity in the gut microbiomes of herbivores/omnivores, zooplanktivores/zoobenthivores and piscivores identified in the CCA. These 35 ASV indicators included 15 indicators for herbivores/omnivores (IV ≥ 0.28, *p* ≤ .01), 12 indicators for zooplanktivores/zoobenthivores (IV ≥ 0.4, *p* ≤ .01), and 8 indicators for piscivores (IV ≥ 0.2, *p* ≤ .01) (Table S3). The three feeding habits featured different bacteria; for example, (1) herbivore/omnivore indicators mainly included *Erysipelotrichaceae*, Mollicutes, *Arcobacter* and *PW3*; (2) zooplanktivore/zoobenthivore indicators were mainly composed of *Hyphomicrobiaceae*, *Desulfobulbaceae*, *Microthrixaceae* and Ellin6529; and (3) the indicators for piscivores primarily consisted of *Acinetobacter johnsonii*, *Photobacterium damselae* and *Shewanella*. The gut microbiomes of fish with these three feeding habits had characteristic fractions of the corresponding indicator bacteria that were significantly higher than those associated with the other feeding habit groups (Figure 3c). It was also noted that some of the most abundant indicators, such as *Erysipelotrichaceae* in herbivores/omnivores and *Photobacterium* in piscivores, were also the dominant groups associated with the corresponding feeding habit, indicating their key roles in shaping the gut microbial communities.

### 3.4 Bacterial diversity couples with trophic adaptation

Although the evaluation of fish feeding habits based on a literature review of feeding ecology can provide a rough classification scheme, the identification of trophic level according to the stable isotope of nitrogen in the fish tissues may more precisely reflect the trophic position and feeding habit of each fish species. In general, $\delta^{15}$δ15N is transferred from prey to predator, resulting in stepwise enrichment with an increase in trophic position. In practice, the measured trophic levels of individual fish species in this study were not entirely consistent with their feeding habit patterns as suggested by the literature. For example, the average trophic level of the herbivore *Siganus canaliculatus* was higher than that of the zoobenthivore/piscivore *Inegocia japonica* (Figure S6), rationalizing the analysis of the gut microbiota from the point of view of the actual trophic position. According to their calculated trophic levels, the 20 fish species in this study were classified into four groups (<3, 3–3.3, 3.3–3.4 and >3.4, corresponding to Levels 1, 2, 3 and 4 consumers, respectively) (Table S4). Permutation analysis indicated that the contribution of the trophic level group to gut microbiome variation was significant, although trophic level did not appear to be a strong determinant (Table S5, *p* < .01, *R^2^* = 0.04, *ω* ^2^ = 0.01). The diversity of the fish gut microbiomes was found to slightly decrease as the trophic level increased (Figure S7a). This variation trend is in accordance with previous studies on mammals showing that herbivores host more diverse gut bacteria than omnivores and carnivores because of the need to digest cellulose (Ley et al., 2008). The bacterial communities were generally clustered among fish species according to their trophic level, while the bacterial communities hosted by several fishes at trophic level 2 were distributed near those hosted by fishes at trophic level 3. The CCA ordination results based on gut ASVs also displayed an obvious clustering pattern associated with trophic level groups, with variability explanation percentages of 41%, 33% and 25% by CCA axis 1, 2 and 3, respectively (Figure S7b, Bray–Curtis distance; PERMANOVA, *p* < .001; PERMDISP, *p* > .05). Twenty discriminative ASVs were selected as indicators, primarily comprising *Erysipelotrichaceae* for Level 1 consumers, *Clostridium perfringens* and Sva0725 (in phylum Acidobacteria) for Level 2 consumers, *Desulfovibrionaceae* for Level 3 consumers and *Enterobacteriaceae* for Level 4 consumers (Figure S7c and Table S6).

### 3.5 The core gut microbiomes in the fish gut and common habitats of these core microbiomes

**FIGURE 4:**
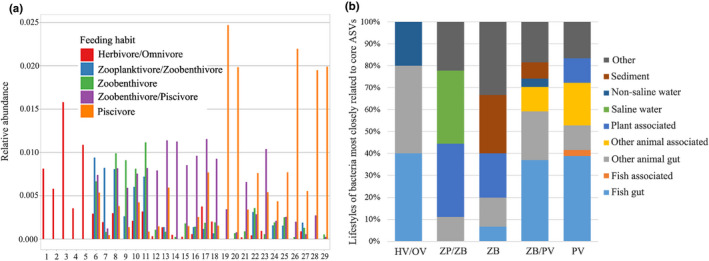
The core gut microbiomes in the fish gut and common habitats of these core microbiomes. (a) Relative abundances of twenty‐nine core ASVs corresponding to the five feeding habits. Core ASVs were separately identified for each feeding habit based on abundance and prevalence. Detailed information about the core microbiome ASVs is provided in Table S7. (b) Closest relatives of core bacterial ASVs. Each core ASV was compared by BLASTn against the NCBI nonredundant nucleotide database, and its closest hit was categorized according to the isolation origins. The proportions of these categories are shown [Colour figure can be viewed at wileyonlinelibrary.com]

Zooplanktivores/zoobenthivores, zoobenthivores and zoobenthivores/piscivores, respectively, contained two, two and five core ASVs (most abundant and prevalent), whereas herbivore/omnivore and piscivore fish did not show a clear core set of ASVs because of the high level of divergence between the most abundant and the most prevalent ASVs (Figure S8). Most of these core gut microbes were particular to the corresponding feeding habits, in which such microbes were much more abundant than in those in fishes with other feeding habits (Figure 4a). Twenty‐five out of twenty‐nine of these abundant or prevalent microbiota were not shared by fishes with different feeding habits, indicating that the gut microbiomes had a high dependency on the host's feeding habit. Among the four shared ASVs, two are classified as members of the genus *Photobacterium* and are shared by zoobenthivore/piscivore and piscivore fish, another two are classified as members of *Ralstonia* and are shared by zoobenthivore and zoobenthivore/piscivore fish, and one belongs to the ML635J‐21 clade and is shared by zooplanktivore/zoobenthivore, zoobenthivore and zoobenthivore/piscivore fish. Among the 25 ASVs, certain populations were enriched in different feeding habit categories, including (1) *Erysipelotrichaceae* and *Desulfovibrionaceae* in the herbivore/omnivore fish, (2) *Ralstonia* and *Gaiellales* in the zooplanktivore/zoobenthivore fish, (3) *Ralstonia* and ML635J‐21 in the zoobenthivore fish, (4) *Photobacterium* in the zoobenthivore/piscivore fish and (5) *Clostridium* and *Cetobacterium* in the piscivore fish.

By analysing the best BLASTn hit of each of the 29 core ASVs (Figure 4b) to explore the common lifestyles of these core gut bacteria, approximately 40% of the hits of the herbivores/omnivores, zoobenthivores/piscivores and piscivores were found to match with the bacteria derived from the fish gut samples. The other hits of the herbivores/omnivores matched other animal gut (40%) and nonsaline water samples (20%). In the zooplanktivores/zoobenthivores, 66% of the total hits were bacteria from plant‐associated samples and saline water, and approximately 10% of the hits were related to the animal gut. Some core microbiota in the zoobenthivores and zoobenthivores/piscivores were found to be similar to those present in sediment, while other animal‐associated bacteria existed in zoobenthivores/piscivores and piscivores. Across all fish specimens, approximately 60% of the core ASVs were most similar to bacteria from animal‐related samples, including those of the fish gut, fish tissues and other animal tissues, such as the gut and skin surface. A small percentage (10%) of the most closely related bacteria existed in plant‐associated samples, and the other 10% were from natural environments, including fresh water, saline water and sediments. These results are partially in accordance with the observation for the Trinidadian guppies(*Poecilia reticulata*) gut microbiome that approximately 60% of the core gut ASVs were also found in animal‐associated samples and that the percentage was 30–40% for environmental samples (Sullam et al., 2015). In terms of the different feeding habits, the core gut microbiomes of fish tend to be enriched in taxa concurrently inhabiting the same environment in addition to the fish gut and associated tissues. For instance, the zoobenthivores and zoobenthivores/piscivores harboured gut microbiota similar to those from sediment, while piscivorous fishes hosted bacteria observed in other animal‐associated tissues and water. These findings generally support the view that the ambient environment is a source from which fishes acquire their gut microbiota.

### 3.6 Predicted fish gut microbiota function and digestion‐related bacteria analysis

Here, whether an increased ASV diversity confers the host with a higher functional diversity was addressed. From the predicted metagenomes, we identified a total of 7,642 KEGG orthologs (KOs), corresponding to 294 KEGG L3 pathways, 40 KEGG L2 pathways and 6 KEGG L1 pathways (Figure S9). In general, the KEGG pathways showed a similar distribution within the different fish species in both pathway level 1 and level 2, and clustering patterns of the fish species based on pathway levels 1 and 2 were observed as well.

**FIGURE 5:**
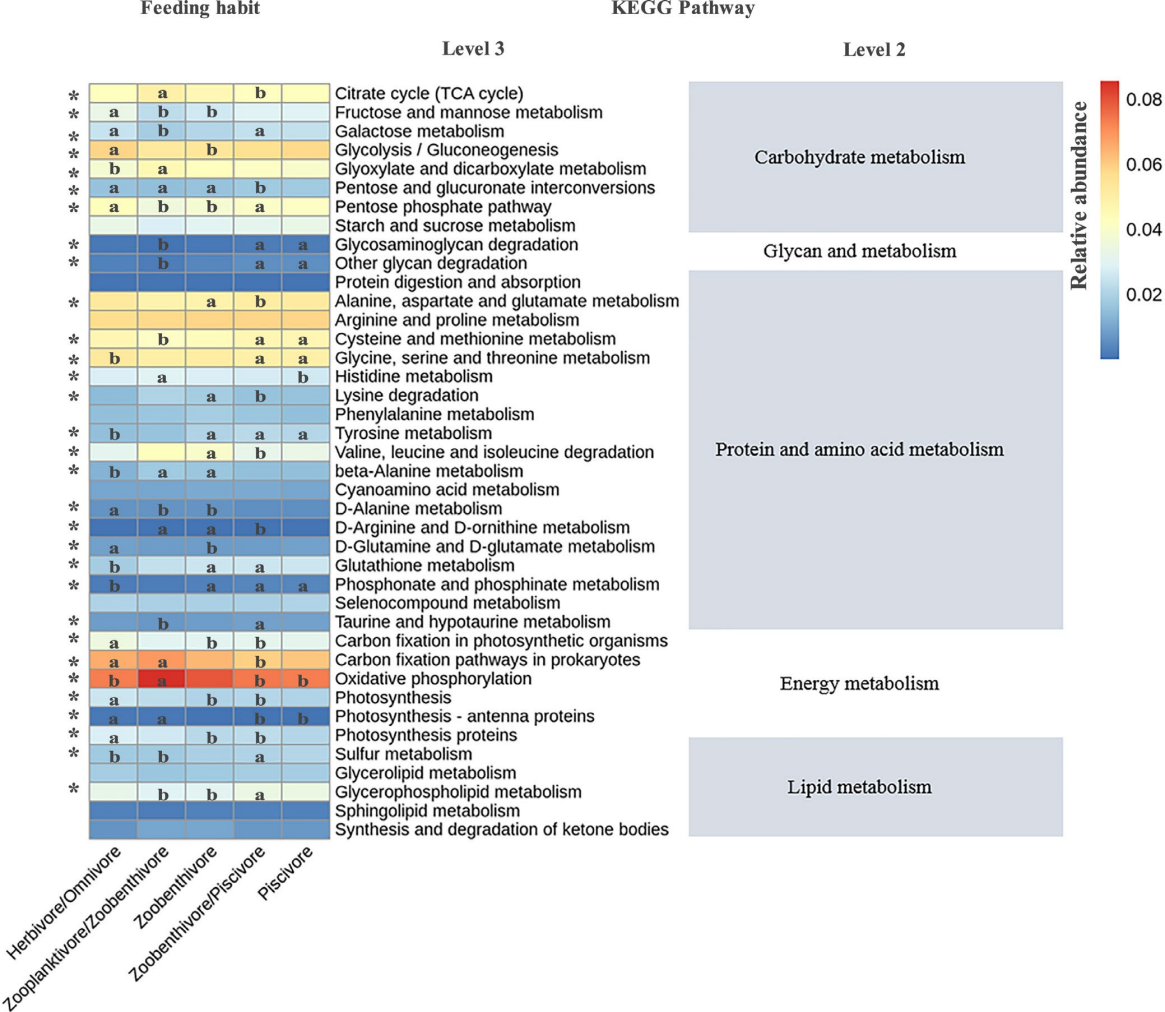
Heatmap presenting the abundance of digestion‐related bacterial gene functions among the five feeding habits. Samples marked by an asterisk (*) indicate significant differences (*p* < .05) among the feeding habits [Colour figure can be viewed at wileyonlinelibrary.com]

Forty pathways related to digestion were identified, including those associated with carbohydrate, glycan, protein and amino acid, energy and lipid metabolism, of which 31 pathways exhibited significant differences in abundance among the five feeding habits (Figure 5). Some pathways related to energy metabolism (i.e. carbon fixation in photosynthetic organisms, photosynthesis, photosynthesis‐antenna proteins, oxidative phosphorylation, carbon fixation pathways in prokaryotes) were enriched in herbivores/omnivores and zooplanktivores/zoobenthivores, whereas pathways related to lipid metabolism (sulphur metabolism and glycerophospholipid metabolism) were enriched in zoobenthivores/piscivores. Glycan metabolism was enriched in both zoobenthivores/piscivores and piscivores. Some of the protein and amino acid metabolism pathways (i.e. phosphonate, phosphinate, glutathione, cysteine, methionine, clycine, serine, threonine and tyrosine pathways) were highly abundant in zoobenthivores, zoobenthivores/piscivores and piscivores. For some pathways related to carbohydrate metabolism, glyoxylate and dicarboxylate metabolism was more enriched in zooplanktivores/zoobenthivores than in herbivores/omnivores, while fructose, mannose, galactose, glycolysis/gluconeogenesis, pentose, glucuronate interconversions and pentose phosphate were more enriched in herbivores/omnivores.

To better understand the relationship between digestion‐related genes and the fish gut microbiota, ASVs significantly correlated with digestion genes in particular were identified by hierarchical all‐against‐all association (HAIIA) (Rahnavard et al.) (Figure S10). This analysis showed that the associated ASVs belong to several specific bacterial taxa, including *Photobacterium,* ML635J‐21 class, *Ralstonia* and *Acinetobacter guillouiae. Photobacterium* exhibits a distinct positive correlation with glycosaminoglycan degradation and other glycan degradation, while and *Acinetobacter guillouiae* was found to be negatively correlated with photosynthesis and photosynthesis proteins pathway. *Ralstonia* and ML635J‐21 class were strongly and positively correlated with 12 pathways (e.g. protein digestion and absorption, phenylalanine metabolism, synthesis and degradation of ketone bodies and lysine degradation). *Photobacterium*, *Ralstonia* and ML635J‐21 were three of the most dominant bacterial taxa across the surveyed fish gut communities in this study. In a study of Atlantic cod gut microbiomes, *Photobacterium iliopiscarium* and *Photobacterium kishitanii* were found to be particularly abundant (Riiser et al., 2019). *Photobacterium iliopiscarium* is a bioluminescent bacterium known for containing the lux‐rib operon, and *Photobacterium kishitanii* is nonluminous, but its functional role in the intestine is still unclear (Riiser et al., 2019). *Ralstonia* is a gram‐negative bacterial genus including nonfermentative Bacilli ubiquitously found in the environment (Ryan et al., 2011) and in sea bass (Carda‐Dieguez et al., 2014), yellow catfish (Shangong Wu et al., 2010) and rainbow trout (Kim et al., 2007). The most common bacterium species of *Ralstonia*, *Ralstonia pickettii*, is an important human pathogen that causes infections such as osteomyelitis and meningitis and may become a threat to seafood safety (Ryan & Adley, 2014).

### 3.7 Comparison of fish and other animal gut microbiomes

**FIGURE 6:**
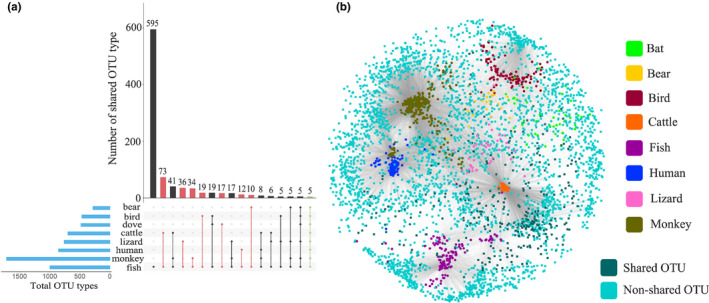
Comparison of fish and other animal gut microbiomes. (a) Intersecting gut microbiomes of fish and other animals (bars in pink represent OTUs uniquely shared by fish and one another animal; the green bar represents OTUs shared by all the animals). (b) Network analysis of gut microbiomes derived from fish and other animals. Dark and light green nodes correspond to shared OTUs and nonshared OTUs, respectively, between fish and other animals (97% similarity) [Colour figure can be viewed at wileyonlinelibrary.com]

A total of 3,540 OTUs (97% similarity) were picked from the publicly available data sets of animal gut microbiomes and the fish gut microbiome in this study by closed‐reference clustering against the Greengenes 13.8 database, among which 999 OTUs were observed in fish gut microbiomes (Figure 6a). The gut microbiomes of fish were quite distinct from those of other animals since only approximately 10% (369) of OTUs were shared by fish and other animals (Figure 6b). Among these 10% shared OTUs, the fish shared 173 (4.9%) OTUs with monkeys and 138 (4.0%) with lizards, accounting for 12% and 11% of the total OTU abundance in fish, respectively. Bears, doves, humans and birds shared 100 (2.8%), 89 (2.5%), 66 (1.9%) and 61 (1.7%) OTUs with fish, respectively, and the corresponding OTU abundances were 4.9%, 4.5%, 1.9% and 4.3%. Furthermore, the 153 (4.3%) OTUs shared with cattle only accounted for 1% of the total OTU abundance in fish. Notably, only 5 OTUs were detected in all of the studied animals, which belong to three families: *Microbacteriaceae*, *Enterobacteriaceae* and *Peptostreptococcaceae*. *Microbacteriaceae* was not commonly reported in the fish gut samples but was found in the intestine of xylophagous beetle larvae (Mohammed et al., 2018). *Enterobacteriaceae* is a taxonomically diverse bacterial family associated with pathogenesis and virulence (Martinson et al., 2019). *Peptostreptococcaceae* is also a common commensal bacterial taxon and may play a role in maintaining gut homeostasis because of its higher abundance in healthy animals than in ill animals (Fan et al., 2017).

In addition, over half of the OTUs (595/999) in the fish samples were uniquely to the fish samples without being detected in other animal samples (Figure 6a). The number of unique intersecting OTUs between fish and one kind of other animal (only existing in these two sets but not in all other animals) decreased from the fish‐cattle (73 OTUs), fish‐lizard (36 OTUs), fish‐monkey (34 OTUs), fish‐dove (17 OTUs), fish‐human (12 OTUs) to fish‐bear (10 OTUs) associations. These results demonstrate the distinction among the gut microbial communities of animals. Similar findings have been reported showing that human gut microbiomes share overlapping gene catalogues with animals at low rates, that is 3.2% with dogs, 8.2% with pigs and 1.2% with mice (Coelho et al., 2018).

## 4 DISCUSSION

Uncovering the patterns of gut microbial communities adjusted by different factors is fundamental for improving host physiological performance, and identifying the dominant fish gut microbiome is one of the basic foci. In many fish species, Proteobacteria, Bacteroidetes and Firmicutes comprise a large proportion of the gut microbiota (Ghanbari et al., 2015). Nielsen et al. (2017) found that the dominant gut microbiome phyla of *Siganus fuscescens* include Proteobacteria, Cyanobacteria and Firmicutes. Dehler et al. (2017) discovered that Proteobacteria and Firmicutes are the most abundant phyla in *Salmo salar* gut microbiomes. Proteobacteria and Planctomycetaceae are dominant among *Gambusia affinis* gut microbiomes according to Carlson et al. (2017). *Danio rerio* (zebrafish), a model organism for the study of genes and pathways related to development, metabolism and disease (Sprague et al., 2006), harbours Proteobacteria, Firmicutes, Fusobacteria, Actinobacteria and Bacteroidetes as the dominant phyla in its intestine (Stephens et al., 2016). In *Salvelinus alpinus* (Arctic char), Proteobacteria, Firmicutes, Actinobacteria and Bacteroidetes are the most abundant phyla (Nyman et al., 2017), while Proteobacteria, Firmicutes and Bacteroidetes are dominant in *Oncorhynchus mykiss* (rainbow trout) (Michl et al., 2017). In our study, Proteobacteria and Firmicutes were observed to be the dominant phyla among the 20 marine fish species, which is consistent with these previous studies, suggesting the commonality of the fish gut community.

Further exploration is essential to determine on the significance of the dominant gut bacteria as the major symbionts in the fish physiology. At the genus level, *Clostridium*, *Photobacterium*, *Ralstonia* and *Acinetobacter* were dominant. Previous studies have revealed that gut bacteria may confer benefits on host digestion, nutrition and immunity. *Clostridium* is a very common group in the animal gut community (Lopetuso et al., 2013). Some species of *Clostridium* could play a role as mutualistic symbionts with hosts, especially with herbivorous fish (Clements et al., 2006). *Clostridium* symbionts in the digestive tract have been shown to contribute to host nutrition, such as supplying fatty acids and vitamins and producing digestive enzymes to degrade cellulose (Liu et al., 2016). *Photobacterium* have been primarily found in the marine environment (Gomez‐Gil et al., 2016); some species of this genus act as mutualistic bacteria in the host gut, aiding with chitin digestion by secreting chitinase (Ray et al., 2012), while others produce harmful excretions and are common pathogens of aquatic animals (Abdel‐Aziz et al., 2019). Regarding *Acinetobacter*, studies have reported that some *Acinetobacter* bacteria carry antibiotic resistance genes (Lee et al., 2017), and some could generate chitinase or lipase to benefit host digestion (Johnson et al., 2015; Kim et al., 2017). Bacteria from the low‐abundance general genera *Propionibacterium*, which are primarily facultative parasites and commensals of humans and other animals and producers of vitamin B12, tetrapyrrole compounds and propionic acid (Piwowarek et al., 2018), were universally found in over 80% of samples in this study with an average abundance of 1.29% of sequences, indicating their important roles in synthesizing useful materials for host fish.

As previously stated, *Siganus canaliculatus*, *Solea ovata* and *Parachaeturichthys polynema* harboured over 70% of unidentified microbes at the genus level. *Siganus canaliculatus* is mainly herbivorous, feeding on benthic algae, and is commonly consumed by humans (Woodland et al., 1990). *Solea ovata* is a flatfish species that mainly feeds on benthic invertebrates, especially crustaceans (Carpenter & Niem, 2001). *Parachaeturichthys polynema* is a zoobenthivore that mainly feeds on benthic crustaceans (Carpenter & Niem, 2001). These three fish species all live near the seabed and feed on benthic organisms, suggesting the uniqueness of their gut microbiomes, which might be associated with the sediment habitat. These unknown microbes suggest the presence of a large knowledge gap and uncertainties in the sequencing analysis of fish gut microbiomes. Therefore, further studies involving the performance of a series of cultivation experiments to uncover the unknown bacteria in these fish species are needed.

The results of our work indicate that the host taxon, feeding habit and trophic level of fish exert significant but partial effects on the host gut microbial communities to varying degrees, that is 16% for host taxon, 6% for feeding habit and 4% for trophic level. The complicated factors (host‐related and environmental factors) influence which bacterium to grow or decline. Thus, developing rankings or quantifying the relative contributions of affecting factors instead of limiting studies to qualification also benefits the exploration about the mechanisms of gut bacteria assembly. Surrounding environments such as water and sediment are considered to be major routes of gut microbiota acquisition and enrichment, as demonstrated in grass carp (Wu et al., 2012) and turbot (Xing et al., 2013). In a previous study on the faecal microbiomes of carp, the environment (wild vs laboratory) was suggested to be a dominant factor shaping the microbial community in their faeces, while diet did not strongly affect the structure in lab‐housed fishes compared with that in wild fishes (Eichmiller et al., 2016). However, some studies suggest that fish gut microbiota are not mainly regulated by the host's ambient environment and that host‐related factors play a dominant role. In a study on African cichlids, diet was demonstrated to drive gut microbiota composition clustering, while the effect of host taxon was significant though relatively small (Baldo et al., 2017). The mechanism of the change occurring in the gut microbial community under the influence of different factors is still unclear and complicated, as distinct variation patterns were observed in all these studies. These findings imply that environmental factors and host‐related factors interact with one another, resulting in complicated outcomes. For example, fish taxonomic identity determines the host's diet habit; therefore, hosts may select the microbiota that are beneficial for digesting the corresponding food sources (e.g. plant vs animal) and assimilating nutrients (e.g. amino acids vs saccharides). Additionally, environmental conditions, especially nutrient status and aquatic community structure, strongly affect the food source diversity and trophic level of the host and subsequently affect the gut microbiome. Host genetic factors are the basis by which the genotype of the gut microbiome is innately encoded, while environmental factors, especially diet, are capable of shifting the gut microbiome postnatally.

Although studies investigating fish gut microbiome composition and diversity have been rapidly increasing in recent years, some research questions require further exploration in the future. Future work should move from describing the gut microbiota taxonomic profile to addressing the functional characterization of the host and manipulation of gut microecology to improve host growth and immune response. Approaches to unravelling the functions of gut microbiota include culture‐dependent methods (i.e. using selective media to isolate pure colonies of bacteria) or culture‐independent methods (e.g. metagenomics, transcriptomics, proteomics, and metabolomics). In our study, the bacteria associated with different feeding habits might serve as potential producers of corresponding enzymes in hosts. More specifically, the herbivore/omnivore‐discriminative ASV Group I bacteria (e.g. *Erysipelotrichaceae*) may contribute to cellulase and amylase production. Likewise, zooplanktivore/zoobenthivore‐discriminative bacteria (e.g. *Microthrixaceae*) may be associated with chitinase, while piscivore‐discriminative bacteria (e.g. *Shewanella*) might be digestive enzyme secretors of lipase, protease and peptidase. In addition, enzyme‐producing bacteria isolated from fish intestines have previously been reported, including *Bacillus*, *Acinetobacter*, *Aeromonas*, *Flavobacterium*, *Photobacterium*, *Pseudomonas*, *Vibrio*, *Microbacterium*, *Micrococcus*, *Staphylococcus* and some unidentified anaerobic bacteria, as potential contributors to amylase, cellulase, protease, lipase, phytase, tannase, xylanase and chitinase production (Ray et al., 2012). The regulation of gut microecology to favour beneficial microbiota by administrating probiotics could increase the generation of metabolites conductive to host physiological activities, and several attempts have been made to investigate the effects of probiotics on fish performance. For example, feeding two lactic acid probiotics, *Lactococcus curvatus* and *Leuconostoc mesenteroides,* to Persian sturgeons can promote their growth rate, viability and digestive enzyme activity (Askarian et al., 2011). In our study, the discriminative bacteria associated with the different feeding habits are postulated to be possible digestion enzyme producers. Additional efforts, such as isolating and cultivating fish gut bacteria, conducting in vitro and in vivo digestion trials with the bacteria, and carrying out omics surveys, could be made to validate the physiological functions of these bacteria. Future studies are recommended to elucidate the functions of the gut microbial community, which will facilitate the identification of suitable probiotics and pave the way to optimizing fish farming practices and enhancing aquaculture yield.

In summary, we conducted a study on the gut microbiome diversity among 20 marine fish species to explore their general relatedness with host taxon, feeding habit and trophic level, trace the common habitats of core gut microbiomes, compare them with other animal gut microbiomes and link gut microbiomes with digestion‐related functions. Proteobacteria and Firmicutes accounted for approximately 70% of the gut microorganisms in these fishes, and *Clostridium*, *Photobacterium*, *Ralstonia* and *Acinetobacter* were dominant genera. The fish gut microbial community is a microorganism reservoir that is worthy of study like other microbial communities, as a large proportion of ASV reads remain unidentified at the species level. Fish taxon, feeding habit and trophic level are significant factors shaping gut microbiomes, although their contributions to the variation in community composition and structure are relatively small. The gut microbial community may not be a direct reflection of host‐related factors and environmental factors but a comprehensive result of the interaction of these factors. An inverse relationship between the diversity of gut microbiomes and trophic level of fishes was shown. The predicted functional pathways of the gut microbiomes revealed that some energy metabolism pathways were enriched in herbivores/omnivores and zooplanktivores/zoobenthivores, lipid metabolism and glycan metabolism pathways tended to be enriched in zoobenthivores/piscivores, and protein and amino acid metabolism pathways were primarily abundant in zoobenthivores, zoobenthivores/piscivores and piscivores. The low proportion of shared OTUs between fish and other animals suggests a large distinction existing in the different animal gut microbiomes. The characterized indicator microbiota of different fish species could be further applied as host‐tracking markers in biodiversity surveys using environmental DNA methods in future studies. The identified discriminative microbiota of the different feeding habits are potential digestive enzyme producers, and future studies are required to unravel and validate the functions of the microbiota for the development of probiotics for the fish farming industry. Our work contributes a unique set of data for further understanding the role of gut microbiota in host metabolism and paves the way for manipulative studies on the application of gut microbiota as probiotics in maricultural practices.

## AUTHORS' CONTRIBUTIONS

K.M.Y.L., T.Z. and Q.H. initiated and designed this study. Q.H., R.C.T.S., Y.D., Y.P.M. and C.X.W. conducted the sampling, laboratory work and data analyses. Q.H. drafted the main manuscript text. K.M.Y.L. and T.Z. supervised the work and contributed to the data analysis and interpretation as well as the manuscript preparation. All authors reviewed the manuscript.

## Supporting information

Figs S1‐S10

Tables S1–S7

Appendix S1

## Data Availability

The data sets supporting the conclusions of this article were deposited into the NCBI Sequence Read Archive (SRA) database (BioProject ID: PRJNA615125). The R scripts and relevant data for data analysis and plotting are available at https://github.com/QiHUANG07/Fish_gut_microbiomes.

## References

[mec15699-bib-0001] Abdel‐Aziz, M. , Eissa, A. E. , Hanna, M. , & Okada, M. A. (2019). Identifying some pathogenic Vibrio/Photobacterium species during mass mortalities of cultured Gilthead seabream (*Sparus aurata*) and European seabass (*Dicentrarchus labrax*) from some Egyptian coastal provinces. International Journal of Veterinary Sciences and Medicine, 1(2), 87–95. 10.1016/j.ijvsm.2013.10.004

[mec15699-bib-0002] Anderson, M. , Gorley, R. N. , & Clarke, K. (2008). PERMANOVA+ for primer: Guide to software and statistical methods. Plymouth: PRIMER‐E.

[mec15699-bib-0003] Askarian, F. , Kousha, A. , Salma, W. , & RingØ, E. (2011). The effect of lactic acid bacteria administration on growth, digestive enzyme activity and gut microbiota in Persian sturgeon *(Acipenser persicus*) and beluga (*Huso huso*) fry. Aquaculture Nutrition, 17(5), 488–497. 10.1111/j.1365-2095.2010.00826.x

[mec15699-bib-0004] Baldo, L. , Pretus, J. L. , Riera, J. L. , Musilova, Z. , Bitja Nyom, A. R. , & Salzburger, W. (2017). Convergence of gut microbiotas in the adaptive radiations of African cichlid fishes. ISME Journal, 11(9), 1975–1987. 10.1038/ismej.2017.62 PMC5560477 28509910

[mec15699-bib-0005] Bates, J. M. , Akerlund, J. , Mittge, E. , & Guillemin, K. (2007). Intestinal alkaline phosphatase detoxifies lipopolysaccharide and prevents inflammation in zebrafish in response to the gut microbiota. Cell Host & Microbe, 2(6), 371–382. 10.1016/j.chom.2007.10.010 18078689 PMC2730374

[mec15699-bib-0006] Bates, J. M. , Mittge, E. , Kuhlman, J. , Baden, K. N. , Cheesman, S. E. , & Guillemin, K. (2006). Distinct signals from the microbiota promote different aspects of zebrafish gut differentiation. Developmental Biology, 297(2), 374–386. 10.1016/j.ydbio.2006.05.006 16781702

[mec15699-bib-0007] Butterfield, N. J. , Knoll, A. H. , & Swett, K. (1990). A bangiophyte red alga from the Proterozoic of arctic Canada. Science, 250, 104–107. 10.1126/science.11538072 11538072

[mec15699-bib-0008] Caicedo, H. H. , Hashimoto, D. A. , Caicedo, J. C. , Pentland, A. , & Pisano, G. P. (2020). Overcoming barriers to early disease intervention. Nature Biotechnology, 38(6), 669–673. 10.1038/s41587-020-0550-z 32444852

[mec15699-bib-0009] Callahan, B. J. , McMurdie, P. J. , Rosen, M. J. , Han, A. W. , Johnson, A. J. , & Holmes, S. P. (2016). DADA2: High‐resolution sample inference from Illumina amplicon data. Nature Methods, 13(7), 581–583. 10.1038/nmeth.3869 27214047 PMC4927377

[mec15699-bib-0010] Caporaso, J. G. , Kuczynski, J. , Stombaugh, J. , Bittinger, K. , Bushman, F. D. , Costello, E. K. , Fierer, N. , Peña, A. G. , Goodrich, J. K. , Gordon, J. I. , Huttley, G. A. , Kelley, S. T. , Knights, D. , Koenig, J. E. , Ley, R. E. , Lozupone, C. A. , McDonald, D. , Muegge, B. D. , Pirrung, M. , … Knight, R. (2010). QIIME allows analysis of high‐throughput community sequencing data. Nature Methods, 7(5), 335–336. 10.1038/nmeth.f.303 20383131 PMC3156573

[mec15699-bib-0011] Carda‐Dieguez, M. , Mira, A. , & Fouz, B. (2014). Pyrosequencing survey of intestinal microbiota diversity in cultured sea bass (*Dicentrarchus labrax*) fed functional diets. FEMS Microbiology Ecology, 87(2), 451–459. 10.1111/1574-6941.12236 24730648

[mec15699-bib-0012] Carlson, J. M. , Leonard, A. B. , Hyde, E. R. , Petrosino, J. F. , & Primm, T. P. (2017). Microbiome disruption and recovery in the fish Gambusia affinis following exposure to broad‐spectrum antibiotic. Infection and Drug Resistance, 10, 143–154. 10.2147/IDR.S129055 28533691 PMC5431701

[mec15699-bib-0013] Carpenter, K. E. , & Niem, V. H. (2001). FAO species identification guide for fishery purposes. The living marine resources of the Western Central. FAO.

[mec15699-bib-0014] Cheesman, S. E. , Neal, J. T. , Mittge, E. , Seredick, B. M. , & Guillemin, K. (2011). Epithelial cell proliferation in the developing zebrafish intestine is regulated by the Wnt pathway and microbial signaling via Myd88. Proceedings of the National Academy of Sciences, 108(Suppl_1), 4570–4577. 10.1073/pnas.1000072107 PMC3063593 20921418

[mec15699-bib-0015] Clements, K. D. , Angert, E. R. , Montgomery, W. L. , & Choat, J. H. (2014). Intestinal microbiota in fishes: What's known and what's not. Molecular Ecology, 23(8), 1891–1898. 10.1111/mec.12699 24612310

[mec15699-bib-0016] Clements, K. D. , Pasch, I. B. Y. , Moran, D. , & Turner, S. J. (2006). Clostridia dominate 16S rRNA gene libraries prepared from the hindgut of temperate marine herbivorous fishes. Marine Biology, 150(6), 1431–1440. 10.1007/s00227-006-0443-9

[mec15699-bib-0017] Coelho, L. P. , Kultima, J. R. , Costea, P. I. , Fournier, C. , Pan, Y. , Czarnecki‐Maulden, G. , Hayward, M. R. , Forslund, S. K. , Schmidt, T. S. B. , Descombes, P. , Jackson, J. R. , Li, Q. , & Bork, P. (2018). Similarity of the dog and human gut microbiomes in gene content and response to diet. Microbiome, 6(1), 72 10.1186/s40168-018-0450-3 29669589 PMC5907387

[mec15699-bib-0018] Colwell, R. R. (1962). The bacterial flora of puget sound fish. Journal of Applied Microbiology, 25(2), 147–158. 10.1111/j.1365-2672.1962.tb01131.x

[mec15699-bib-0019] Conway, J. R. , Lex, A. , & Gehlenborg, N. (2017). UpSetR: An R package for the visualization of intersecting sets and their properties. Bioinformatics, 33(18), 2938–2940. 10.1093/bioinformatics/btx364 28645171 PMC5870712

[mec15699-bib-0020] De Filippo, C. , Cavalieri, D. , Di Paola, M. , Ramazzotti, M. , Poullet, J. B. , Massart, S. , Collini, S. , Pieraccini, G. , & Lionetti, P. (2010). Impact of diet in shaping gut microbiota revealed by a comparative study in children from Europe and rural Africa. Proceedings of the National Academy of Sciences of the United States of America, 107(33), 14691–14696. 10.1073/pnas.1005963107 20679230 PMC2930426

[mec15699-bib-0021] Dehler, C. E. , Secombes, C. J. , & Martin, S. A. (2017). Environmental and physiological factors shape the gut microbiota of Atlantic salmon parr (*Salmo salar* L.). Aquaculture, 467, 149–157. 10.1016/j.aquaculture.2016.07.017 28111483 PMC5142738

[mec15699-bib-0022] DeSantis, T. Z. , Hugenholtz, P. , Larsen, N. , Rojas, M. , Brodie, E. L. , Keller, K. , Huber, T. , Dalevi, D. , Hu, P. , & Andersen, G. L. (2006). Greengenes, a chimera‐checked 16S rRNA gene database and workbench compatible with ARB. Applied and Environmental Microbiology, 72(7), 5069–5072. 10.1128/AEM.03006-05 16820507 PMC1489311

[mec15699-bib-0023] Dhanasiri, A. K. , Brunvold, L. , Brinchmann, M. F. , Korsnes, K. , Bergh, O. , & Kiron, V. (2011). Changes in the intestinal microbiota of wild Atlantic cod *Gadus morhua* L. upon captive rearing. Microbial Ecology, 61(1), 20–30. 10.1007/s00248-010-9673-y 20424834

[mec15699-bib-0024] Egerton, S. , Culloty, S. , Whooley, J. , Stanton, C. , & Ross, R. P. (2018). The gut microbiota of marine fish. Frontiers in Microbiology, 9, 873 10.3389/fmicb.2018.00873 29780377 PMC5946678

[mec15699-bib-0025] Eichmiller, J. J. , Hamilton, M. J. , Staley, C. , Sadowsky, M. J. , & Sorensen, P. W. (2016). Environment shapes the fecal microbiome of invasive carp species. Microbiome, 4(1), 44 10.1186/s40168-016-0190-1 27514729 PMC4981970

[mec15699-bib-0026] Fan, P. , Liu, P. , Song, P. , Chen, X. , & Ma, X. (2017). Moderate dietary protein restriction alters the composition of gut microbiota and improves ileal barrier function in adult pig model. Scientific Reports, 7, 43412 10.1038/srep43412 28252026 PMC5333114

[mec15699-bib-0027] Gatesoupe, F.‐J. , Huelvan, C. , Le Bayon, N. , Le Delliou, H. , Madec, L. , Mouchel, O. , Quazuguel, P. , Mazurais, D. , & Zambonino‐Infante, J.‐L. (2016). The highly variable microbiota associated to intestinal mucosa correlates with growth and hypoxia resistance of sea bass, *Dicentrarchus labrax*, submitted to different nutritional histories. BMC Microbiology, 16(1), 266 10.1186/s12866-016-0885-2 27821062 PMC5100225

[mec15699-bib-0028] Ghanbari, M. , Kneifel, W. , & Domig, K. J. (2015). A new view of the fish gut microbiome: Advances from next‐generation sequencing. Aquaculture, 448, 464–475. 10.1016/j.aquaculture.2015.06.033

[mec15699-bib-0029] Gomez‐Gil, B. , Roque, A. , Rotllant, G. , Romalde, J. L. , Doce, A. , Eggermont, M. , & Defoirdt, T. (2016). *Photobacterium sanguinicancri* sp. nov. isolated from marine animals. Antonie Van Leeuwenhoek, 109(6), 817–825. 10.1007/s10482-016-0681-x 27048242

[mec15699-bib-0030] Guo, F. , & Zhang, T. (2013). Biases during DNA extraction of activated sludge samples revealed by high throughput sequencing. Applied Microbiology and Biotechnology, 97(10), 4607–4616. 10.1007/s00253-012-4244-4 22760785 PMC3647099

[mec15699-bib-0031] Hovda, M. B. , Fontanillas, R. , McGurk, C. , Obach, A. , & Rosnes, J. T. (2012). Seasonal variations in the intestinal microbiota of farmed Atlantic salmon (*Salmo salar L*.). Aquaculture Research, 43(1), 154–159. 10.1111/j.1365-2109.2011.02805.x

[mec15699-bib-0032] IBM Corp (2013). IBM SPSS Statistics for Windows (Version 22.0), Chicago: . IBM Corp.

[mec15699-bib-0033] Izvekova, G. I. , Izvekov, E. I. , & Plotnikov, A. O. (2007). Symbiotic microflora in fishes of different ecological groups. Biological Bulletin, 34(6), 610–618. 10.1134/s106235900706012x 19768967

[mec15699-bib-0034] Johnson, T. L. , Waack, U. , Smith, S. , Mobley, H. , & Sandkvist, M. (2015). Acinetobacter baumannii Is Dependent on the Type II Secretion System and Its Substrate LipA for Lipid Utilization and In Vivo Fitness. Journal of Bacteriology, 198(4), 711–719. 10.1128/JB.00622-15 26668261 PMC4751819

[mec15699-bib-0035] Kelly, B. J. , Gross, R. , Bittinger, K. , Sherrill‐Mix, S. , Lewis, J. D. , Collman, R. G. , Bushman, F. D. , & Li, H. (2015). Power and sample‐size estimation for microbiome studies using pairwise distances and PERMANOVA. Bioinformatics, 31(15), 2461–2468. 10.1093/bioinformatics/btv183 25819674 PMC4514928

[mec15699-bib-0036] Kim, D. H. , Brunt, J. , & Austin, B. (2007). Microbial diversity of intestinal contents and mucus in rainbow trout (*Oncorhynchus mykiss*). Journal of Applied Microbiology, 102(6), 1654–1664. 10.1111/j.1365-2672.2006.03185.x 17578431

[mec15699-bib-0037] Kim, T. I. , Ki, K. S. , Lim, D. H. , Vijayakumar, M. , Park, S. M. , Choi, S. H. , & Park, B. Y. (2017). Novel Acinetobacter parvus HANDI 309 microbial biomass for the production of N‐acetyl‐beta‐d‐glucosamine (GlcNAc) using swollen chitin substrate in submerged fermentation. Biotechnology for Biofuels, 10, 59 10.1186/s13068-017-0740-1 28293289 PMC5345198

[mec15699-bib-0038] Lee, C.‐R. , Lee, J. H. , Park, M. , Park, K. S. , Bae, I. K. , Kim, Y. B. , Cha, C.‐J. , Jeong, B. C. , & Lee, S. H. (2017). Biology of Acinetobacter baumannii: Pathogenesis, antibiotic resistance mechanisms, and prospective treatment options. Frontiers in Cellular and Infection Microbiology, 7, 55 10.3389/fcimb.2017.00055 28348979 PMC5346588

[mec15699-bib-0039] Ley, R. E. , Hamady, M. , Lozupone, C. , Turnbaugh, P. J. , Ramey, R. R. , Bircher, J. S. , Schlegel, M. L. , Tucker, T. A. , Schrenzel, M. D. , Knight, R. , & Gordon, J. I. (2008). Evolution of mammals and their gut microbes. Science, 320(5883), 1647–1651. 10.1126/science.1155725 18497261 PMC2649005

[mec15699-bib-0040] Ley, R. E. , Lozupone, C. A. , Hamady, M. , Knight, R. , & Gordon, J. I. (2008). Worlds within worlds: Evolution of the vertebrate gut microbiota. Nature Review Microbiology, 6(10), 776–788. 10.1038/nrmicro1978 18794915 PMC2664199

[mec15699-bib-0041] Liu, H. , Guo, X. , Gooneratne, R. , Lai, R. , Zeng, C. , Zhan, F. , & Wang, W. (2016). The gut microbiome and degradation enzyme activity of wild freshwater fishes influenced by their trophic levels. Scientific Reports, 6, 24340 10.1038/srep24340 27072196 PMC4829839

[mec15699-bib-0042] Llewellyn, M. S. , McGinnity, P. , Dionne, M. , Letourneau, J. , Thonier, F. , Carvalho, G. R. , Creer, S. , & Derome, N. (2016). The biogeography of the atlantic salmon (*Salmo salar*) gut microbiome. ISME Journal, 10(5), 1280–1284. 10.1038/ismej.2015.189 PMC5029221 26517698

[mec15699-bib-0043] Lopetuso, L. R. , Scaldaferri, F. , Petito, V. , & Gasbarrini, A. (2013). Commensal Clostridia: Leading players in the maintenance of gut homeostasis. Gut Pathogens, 5(1), 23 10.1186/1757-4749-5-23 23941657 PMC3751348

[mec15699-bib-0044] Lyons, P. P. , Turnbull, J. F. , Dawson, K. A. , & Crumlish, M. (2017). Phylogenetic and functional characterization of the distal intestinal microbiome of rainbow trout Oncorhynchus mykiss from both farm and aquarium settings. Journal of Applied Microbiology, 122(2), 347–363. 10.1111/jam.13347 27860093

[mec15699-bib-0045] Margolis, L. (1953). The effect of fasting on the bacterial flora of the intestine of fish. Journal of the Fisheries Research Board of Canada, 10(2), 62–63. 10.1139/f53-003

[mec15699-bib-0046] Martinson, J. N. V. , Pinkham, N. V. , Peters, G. W. , Cho, H. , Heng, J. , Rauch, M. , Broadaway, S. C. , & Walk, S. T. (2019). Rethinking gut microbiome residency and the Enterobacteriaceae in healthy human adults. ISME Journal, 13(9), 2306–2318. 10.1038/s41396-019-0435-7 PMC6776003 31089259

[mec15699-bib-0047] Michl, S. C. , Ratten, J. M. , Beyer, M. , Hasler, M. , LaRoche, J. , & Schulz, C. (2017). The malleable gut microbiome of juvenile rainbow trout (*Oncorhynchus mykiss*): Diet‐dependent shifts of bacterial community structures. PLoS One, 12(5), e0177735 10.1371/journal.pone.0177735 28498878 PMC5428975

[mec15699-bib-0048] Mohammed, W. S. , Ziganshina, E. E. , Shagimardanova, E. I. , Gogoleva, N. E. , & Ziganshin, A. M. (2018). Comparison of intestinal bacterial and fungal communities across various xylophagous beetle larvae (Coleoptera: Cerambycidae). Scientific Reports, 8(1), 10073 10.1038/s41598-018-27342-z 29968731 PMC6030058

[mec15699-bib-0049] Nayak, S. K. (2010). Role of gastrointestinal microbiota in fish. Aquaculture Research, 41(11), 1553–1573. 10.1111/j.1365-2109.2010.02546.x

[mec15699-bib-0050] Nielsen, S. , Wilkes Walburn, J. , Verges, A. , Thomas, T. , & Egan, S. (2017). Microbiome patterns across the gastrointestinal tract of the rabbitfish *Siganus fuscescens* . PeerJ, 5, e3317 10.7717/peerj.3317 28533966 PMC5437856

[mec15699-bib-0051] Nyman, A. , Huyben, D. , Lundh, T. , & Dicksved, J. (2017). Effects of microbe‐ and mussel‐based diets on the gut microbiota in Arctic charr (*Salvelinus alpinus*). Aquaculture Reports, 5, 34–40. 10.1016/j.aqrep.2016.12.003

[mec15699-bib-0052] Pan, D. , & Yu, Z. (2014). Intestinal microbiome of poultry and its interaction with host and diet. Gut Microbes, 5(1), 108–119. 10.4161/gmic.26945 24256702 PMC4049927

[mec15699-bib-0053] Perkins, M. J. , Mak, Y. K. Y. , Tao, L. S. R. , Wong, A. T. L. , Yau, J. K. C. , Baker, D. M. , & Leung, K. M. Y. (2018). Short‐term tissue decomposition alters stable isotope values and C: N ratio, but does not change relationships between lipid content, C: N ratio, and Deltadelta13C in marine animals. PLoS One, 13(7), e0199680 10.1371/journal.pone.0199680 30020988 PMC6051570

[mec15699-bib-0054] Piwowarek, K. , Lipinska, E. , Hac‐Szymanczuk, E. , Kieliszek, M. , & Scibisz, I. (2018). Propionibacterium spp.‐source of propionic acid, vitamin B12, and other metabolites important for the industry. Applied Microbiology and Biotechnology, 102(2), 515–538. 10.1007/s00253-017-8616-7 29167919 PMC5756557

[mec15699-bib-0055] Rahnavard, G. , Franzosa, E. A. , McIver, L. J. , Schwager, E. , Lloyd‐Price, J. , Weingart, G. , & Huttenhower, C. High‐sensitivity pattern discovery in large multi'omic datasets. An unpublished manuscript. https://huttenhower.sph.harvard.edu/halla 10.1093/bioinformatics/btac232 PMC9235493 35758795

[mec15699-bib-0056] Ray, A. K. , Ghosh, K. , & Ringø, E. (2012). Enzyme‐producing bacteria isolated from fish gut: A review. Aquaculture Nutrition, 18(5), 465–492. 10.1111/j.1365-2095.2012.00943.x

[mec15699-bib-0057] Reed, G. B. , & Spence, C. M. (1929). The intestinal and slime flora of the haddock: A preliminary report. Contributions to Canadian Biology and Fisheries, 4(1), 257–264. 10.1139/f29-019

[mec15699-bib-0058] Rhee, K. J. , Sethupathi, P. , Driks, A. , Lanning, D. K. , & Knight, K. L. (2004). Role of commensal bacteria in development of gut‐associated lymphoid tissues and preimmune antibody repertoire. The Journal of Immunology, 172(2), 1118–1124. 10.4049/jimmunol.172.2.1118 14707086

[mec15699-bib-0059] Riiser, E. S. , Haverkamp, T. H. A. , Varadharajan, S. , Borgan, O. , Jakobsen, K. S. , Jentoft, S. , & Star, B. (2019). Switching on the light: Using metagenomic shotgun sequencing to characterize the intestinal microbiome of Atlantic cod. Environmental Microbiology, 21(7), 2576–2594. 10.1111/1462-2920.14652 31091345

[mec15699-bib-0060] Roeselers, G. , Mittge, E. K. , Stephens, W. Z. , Parichy, D. M. , Cavanaugh, C. M. , Guillemin, K. , & Rawls, J. F. (2011). Evidence for a core gut microbiota in the zebrafish. ISME Journal, 5(10), 1595–1608. 10.1038/ismej.2011.38 PMC3176511 21472014

[mec15699-bib-0061] Ryan, M. P. , & Adley, C. C. (2014). Ralstonia spp.: Emerging global opportunistic pathogens. European Journal of Clinical Microbiology and Infectious Diseases, 33(3), 291–304. 10.1007/s10096-013-1975-9 24057141

[mec15699-bib-0062] Ryan, M. P. , Pembroke, J. T. , & Adley, C. C. (2011). Genotypic and phenotypic diversity of *Ralstonia pickettii* and *Ralstonia insidiosa* isolates from clinical and environmental sources including High‐purity Water. Diversity in *Ralstonia pickettii* . BMC Microbiology, 11, 194 10.1186/1471-2180-11-194 21878094 PMC3175462

[mec15699-bib-0063] Sanders, J. G. , Beichman, A. C. , Roman, J. , Scott, J. J. , Emerson, D. , McCarthy, J. J. , & Girguis, P. R. (2015). Baleen whales host a unique gut microbiome with similarities to both carnivores and herbivores. Nature Communications, 6, 8285 10.1038/ncomms9285 PMC4595633 26393325

[mec15699-bib-0064] Schmidt, V. T. , Smith, K. F. , Melvin, D. W. , & Amaral‐Zettler, L. A. (2015). Community assembly of a euryhaline fish microbiome during salinity acclimation. Molecular Ecology, 24(10), 2537–2550. 10.1111/mec.13177 25819646

[mec15699-bib-0065] Segata, N. , Izard, J. , Waldron, L. , Gevers, D. , Miropolsky, L. , Garrett, W. S. , & Huttenhower, C. (2011). Metagenomic biomarker discovery and explanation. Genome Biology, 12(6), R60 10.1186/gb-2011-12-6-r60 21702898 PMC3218848

[mec15699-bib-0066] Shannon, P. , Markiel, A. , Ozier, O. , Baliga, N. S. , Wang, J. T. , Ramage, D. , & Ideker, T. (2003). Cytoscape: A software environment for integrated models of biomolecular interaction networks. Genome Research, 13(11), 2498–2504. 10.1101/gr.1239303 14597658 PMC403769

[mec15699-bib-0067] Simidu, U. , & Hasuo, K. (1968). Salt dependency of the bacterial flora of marine fish. The Journal of General Microbiology, 52(3), 347–354. 10.1099/00221287-52-3-347

[mec15699-bib-0068] Sprague, J. , Bayraktaroglu, L. , Clements, D. , Conlin, T. , Fashena, D. , Frazer, K. , & Westerfield, M. (2006). The Zebrafish Information Network: The zebrafish model organism database. Nucleic Acids Research, 34(Database issue), D581–585. 10.1093/nar/gkj086 16381936 PMC1347449

[mec15699-bib-0069] Stephens, W. Z. , Burns, A. R. , Stagaman, K. , Wong, S. , Rawls, J. F. , Guillemin, K. , & Bohannan, B. J. (2016). The composition of the zebrafish intestinal microbial community varies across development. ISME Journal, 10(3), 644–654. 10.1038/ismej.2015.140 PMC4817687 26339860

[mec15699-bib-0070] Sullam, K. E. , Essinger, S. D. , Lozupone, C. A. , O’connor, M. P. , Rosen, G. L. , Knight, R. , Kilham, S. S. , & Russell, J. A. (2012). Environmental and ecological factors that shape the gut bacterial communities of fish: A meta‐analysis. Molecular Ecology, 21(13), 3363–3378. 10.1111/j.1365-294X.2012.05552.x 22486918 PMC3882143

[mec15699-bib-0071] Sullam, K. E. , Rubin, B. E. , Dalton, C. M. , Kilham, S. S. , Flecker, A. S. , & Russell, J. A. (2015). Divergence across diet, time and populations rules out parallel evolution in the gut microbiomes of Trinidadian guppies. ISME Journal, 9(7), 1508–1522. 10.1038/ismej.2014.231 PMC4478690 25575311

[mec15699-bib-0072] Tao, L. S. R. , Lui, K. K. Y. , Lau, E. T. C. , Ho, K. K. Y. , Mak, Y. K. Y. , de Mitcheson, Y. S. , & Leung, K. M. Y. (2018). Trawl ban in a heavily exploited marine environment: Responses in population dynamics of four stomatopod species. Scientific Reports, 8(1), 17876 10.1038/s41598-018-35804-7 30552339 PMC6294824

[mec15699-bib-0074] Thompson, L. R. , Sanders, J. G. , McDonald, D. , Amir, A. , Ladau, J. , Locey, K. J. , Prill, R. J. , Tripathi, A. , Gibbons, S. M. , Ackermann, G. , Navas‐Molina, J. A. , Janssen, S. , Kopylova, E. , Vázquez‐Baeza, Y. , González, A. , Morton, J. T. , Mirarab, S. , Zech Xu, Z. , Jiang, L. , … Knight R. (2017). A communal catalogue reveals Earth’s multiscale microbial diversity. Nature, 551(7681), 457–463. 10.1038/nature24621 29088705 PMC6192678

[mec15699-bib-0075] Wang, A. R. , Ran, C. , Ringø, E. , & Zhou, Z. G. (2018). Progress in fish gastrointestinal microbiota research. Reviews in Aquaculture, 10(3), 626–640. 10.1111/raq.12191

[mec15699-bib-0076] Wang, Q. , Garrity, G. M. , Tiedje, J. M. , & Cole, J. R. (2007). Naive Bayesian classifier for rapid assignment of rRNA sequences into the new bacterial taxonomy. Applied and Environmental Microbiology, 73(16), 5261–5267. 10.1128/AEM.00062-07 17586664 PMC1950982

[mec15699-bib-0077] Wei, J. , Guo, X. , Liu, H. , Chen, Y. , & Wang, W. (2018). The variation profile of intestinal microbiota in blunt snout bream (*Megalobrama amblycephala*) during feeding habit transition. BMC Microbiology, 18(1), 99 10.1186/s12866-018-1246-0 30176798 PMC6122550

[mec15699-bib-0078] Wong, S. , & Rawls, J. F. (2012). Intestinal microbiota composition in fishes is influenced by host ecology and environment. Molecular Ecology, 21(13), 3100–3102. 10.1111/j.1365-294x.2012.05646.x 22916346 PMC4846280

[mec15699-bib-0079] Woodland, D. J. , Randall, J. E. , & Museum, B. P. B. (1990). Revision of the fish family Siganidae with descriptions of two new species and comments on distribution and biology. Honolulu, Hawaii: Bernice Pauahi Bishop Museum.

[mec15699-bib-0080] Wu, S. , Gao, T. , Zheng, Y. , Wang, W. , Cheng, Y. , & Wang, G. (2010). Microbial diversity of intestinal contents and mucus in yellow catfish (*Pelteobagrus fulvidraco*). Aquaculture, 303(1–4), 1–7. 10.1016/j.aquaculture.2009.12.025

[mec15699-bib-0081] Wu, S. , Wang, G. , Angert, E. R. , Wang, W. , Li, W. , & Zou, H. (2012). Composition, diversity, and origin of the bacterial community in grass carp intestine. PLoS One, 7(2), e30440 10.1371/journal.pone.0030440 22363439 PMC3282688

[mec15699-bib-0082] Xing, M. , Hou, Z. , Yuan, J. , Liu, Y. , Qu, Y. , & Liu, B. (2013). Taxonomic and functional metagenomic profiling of gastrointestinal tract microbiome of the farmed adult turbot (*Scophthalmus maximus*). FEMS Microbiology Ecology, 86(3), 432–443. 10.1111/1574-6941.12174 23802730

[mec15699-bib-0083] Yan, Q. , Li, J. , Yu, Y. , Wang, J. , He, Z. , Van Nostrand, J. D. , Kempher, M. L. , Wu, L. , Wang, Y. , Liao, L. , Li, X. , Wu, S. , Ni, J. , Wang, C. , & Zhou, J. (2016). Environmental filtering decreases with fish development for the assembly of gut microbiota. Environmental Microbiology, 18(12), 4739–4754. 10.1111/1462-2920.13365 27130138

[mec15699-bib-0084] Yoshimizu, M. , & Kimura, T. (1976). Study on the Intestinal Microflora of Salmonids. Fish Pathology, 10(2), 243–259. 10.3147/jsfp.10.243

[mec15699-bib-0085] Zhang, T. , Shao, M. F. , & Ye, L. (2012). 454 pyrosequencing reveals bacterial diversity of activated sludge from 14 sewage treatment plants. ISME Journal, 6(6), 1137–1147. 10.1038/ismej.2011.188 PMC3358032 22170428

[mec15699-bib-0086] Zhang, Y. , & Alekseyenko, A. V. (2017). Phylogenic inference using alignment‐free methods for applications in microbial community surveys using 16s rRNA gene. PLoS One, 12(11), e0187940 10.1371/journal.pone.0187940 29136663 PMC5685621

[mec15699-bib-0087] Zhang, Z. , Li, D. , Refaey, M. M. , Xu, W. , Tang, R. , & Li, L. (2018). Host age affects the development of southern catfish gut bacterial community divergent from that in the food and rearing water. Frontiers in Microbiology, 9, 495 10.3389/fmicb.2018.00495 29616008 PMC5869207

