# Supplementary material for: Diversity of gut microbiomes in marine fishes is shaped by host‐related factors
Source: Mol Ecol. 2020 Nov 9;29(24):5019–34. doi: 10.1111/mec.15699 (PMC7756402; doi:10.1111/mec.15699)
Supplement: Supplementary file 1 — Figs S1‐S10 [file MEC-29-5019-s001.pdf]

MOLECULAR ECOLOGY

Supplemental Information for:

Diversity of gut microbiomes in marine fishes is shaped by host-related factors

Qi Huang, Ronia C. T. Sham, Yu Deng, Yanping Mao, Chunxiao Wang, Tong Zhang, Kenneth M. Y. Leung

Table of Contents:

**Fig. S1**	Page 2
**Fig. S2**	Page 3
**Fig. S3**	Page 4
**Fig. S4**	Page 5
**Fig. S5**	Page 6
**Fig. S6**	Page 7
**Fig. S7**	Page 8
**Fig. S8**	Page 9
**Fig. S9**	Page 10
**Fig. S10**	Page 11

MOLECULAR ECOLOGY

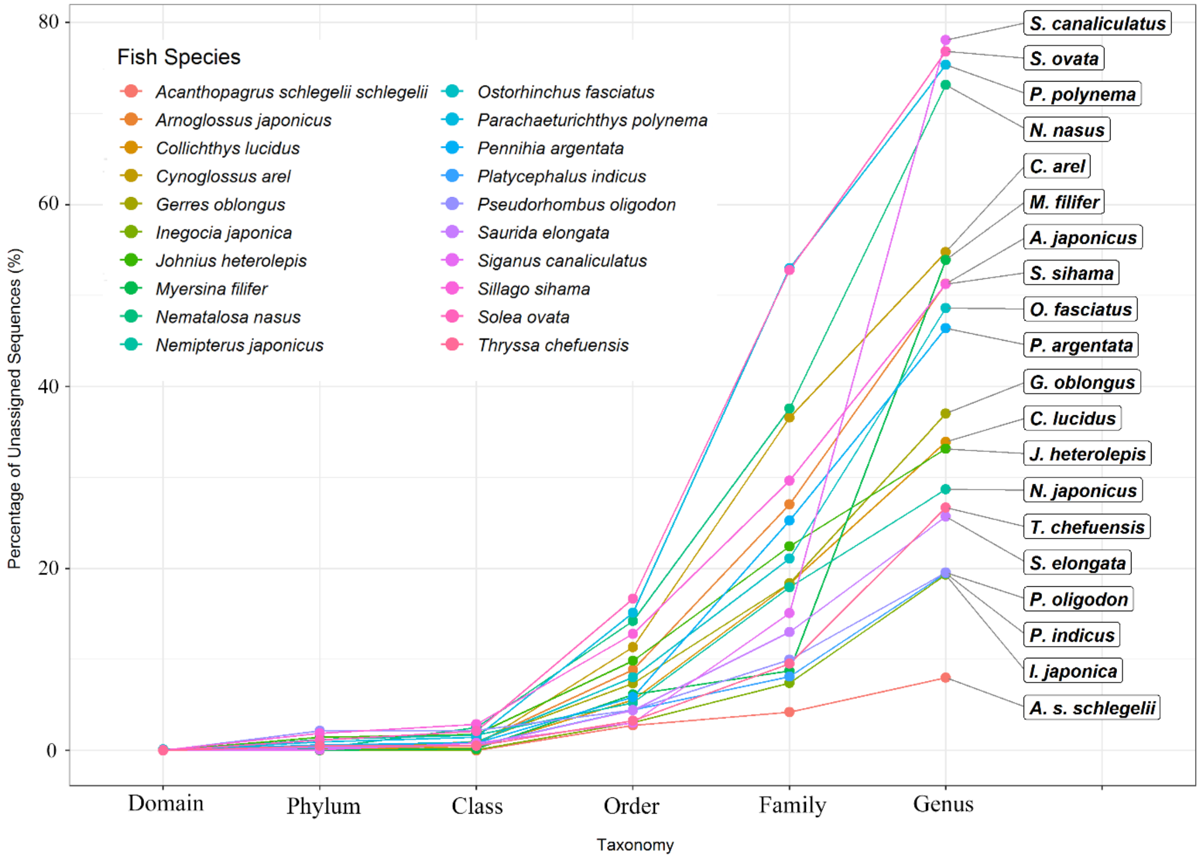

Fig. S1. Percentages of unassigned sequences for fish gut microbiomes at different taxonomic levels. Microbiomes are well annotated at taxonomic levels above the order level, but most remain unknown at the genus level.

MOLECULAR ECOLOGY

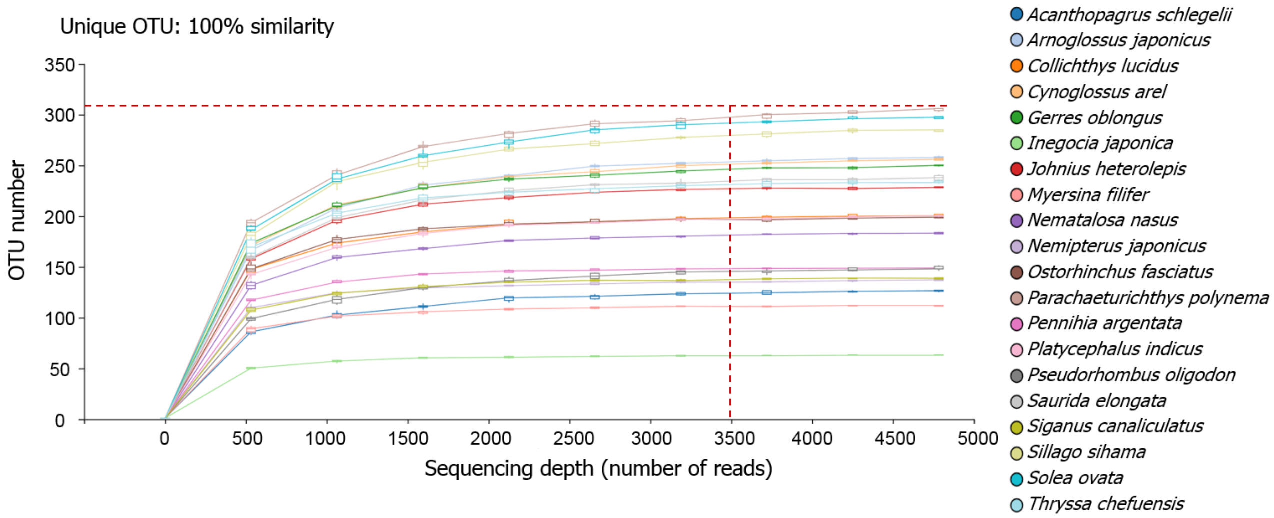

Fig. S2. Cumulative richness of ASVs and sequencing depth. ASV: amplicon sequence variant representing different bacterial individuals. Sequencing depth: number of output reads.

MOLECULAR ECOLOGY

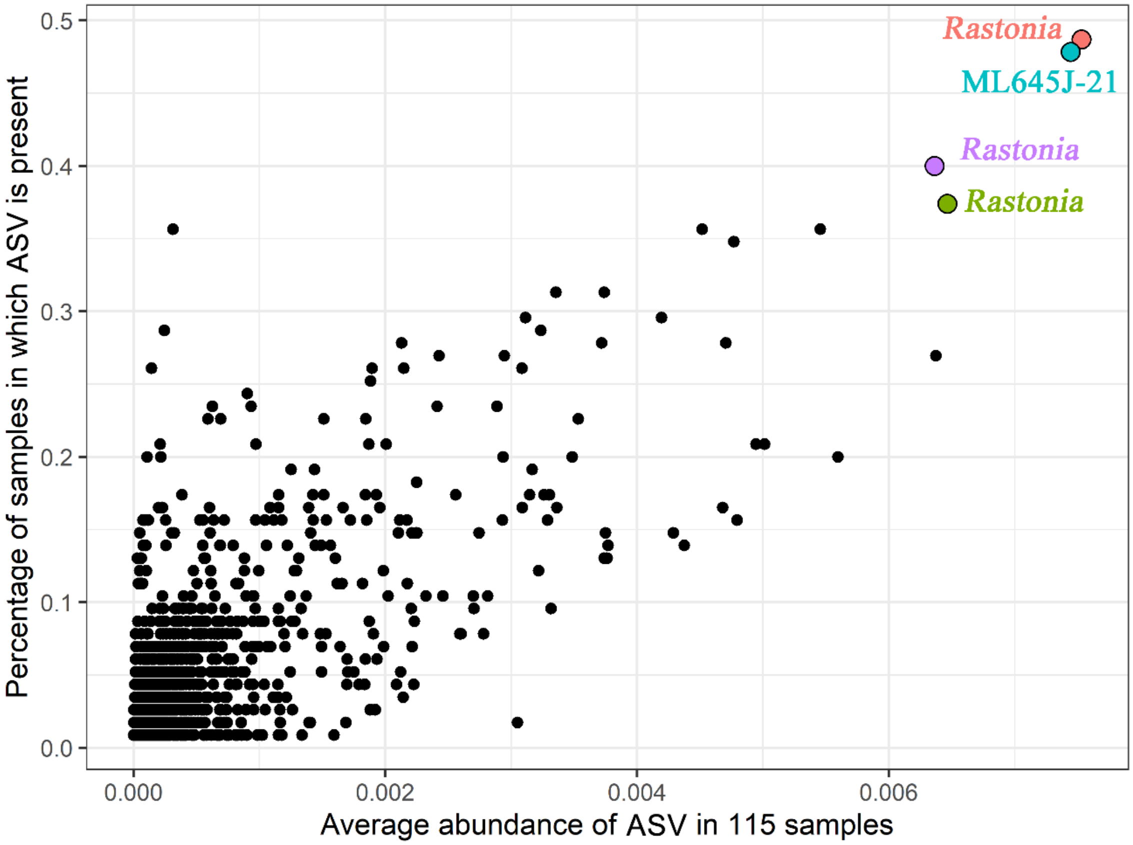

Fig. S3. Abundance against the prevalence of bacteria at the ASV level among the 115 samples. Three of the four dominant ASVs belonged to Ralstonia, and the other ASV belonged to the ML635J-21 class.

MOLECULAR ECOLOGY

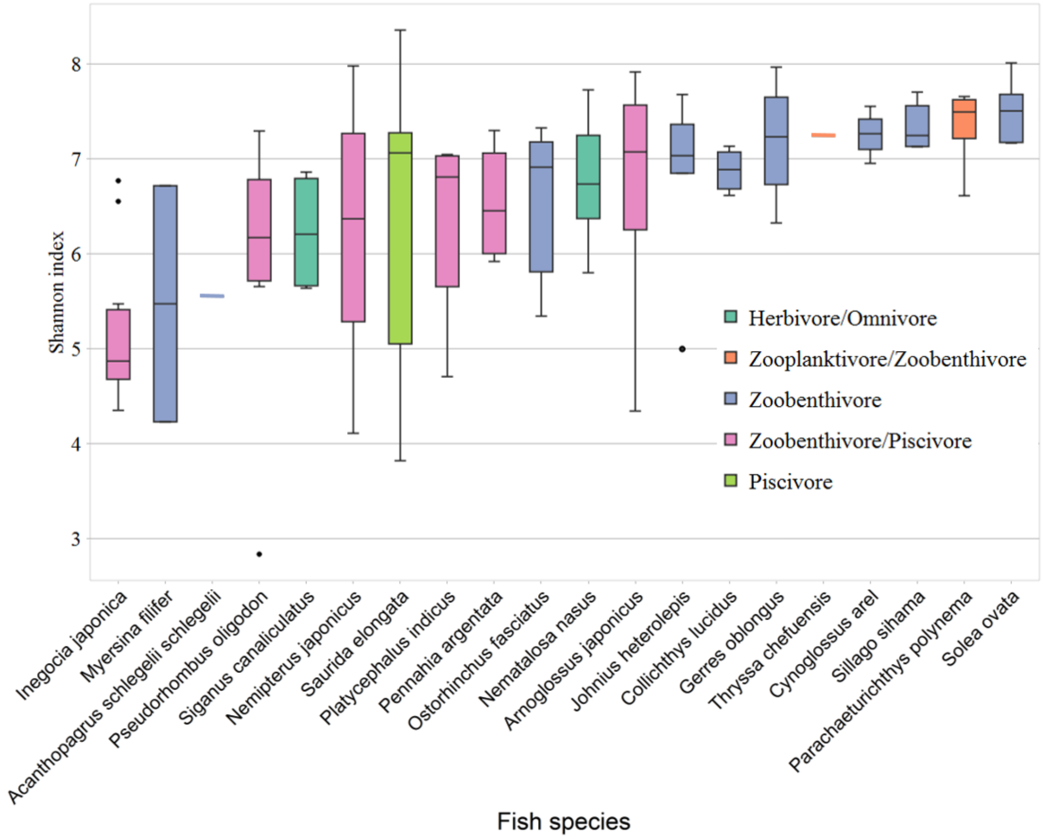

Fig. S4. Box plots of the alpha diversity (Shannon index) of ASVs retrieved from the fish gut microbiome DNA sequences. Different colors represent the different feeding habits of the fishes.

MOLECULAR ECOLOGY

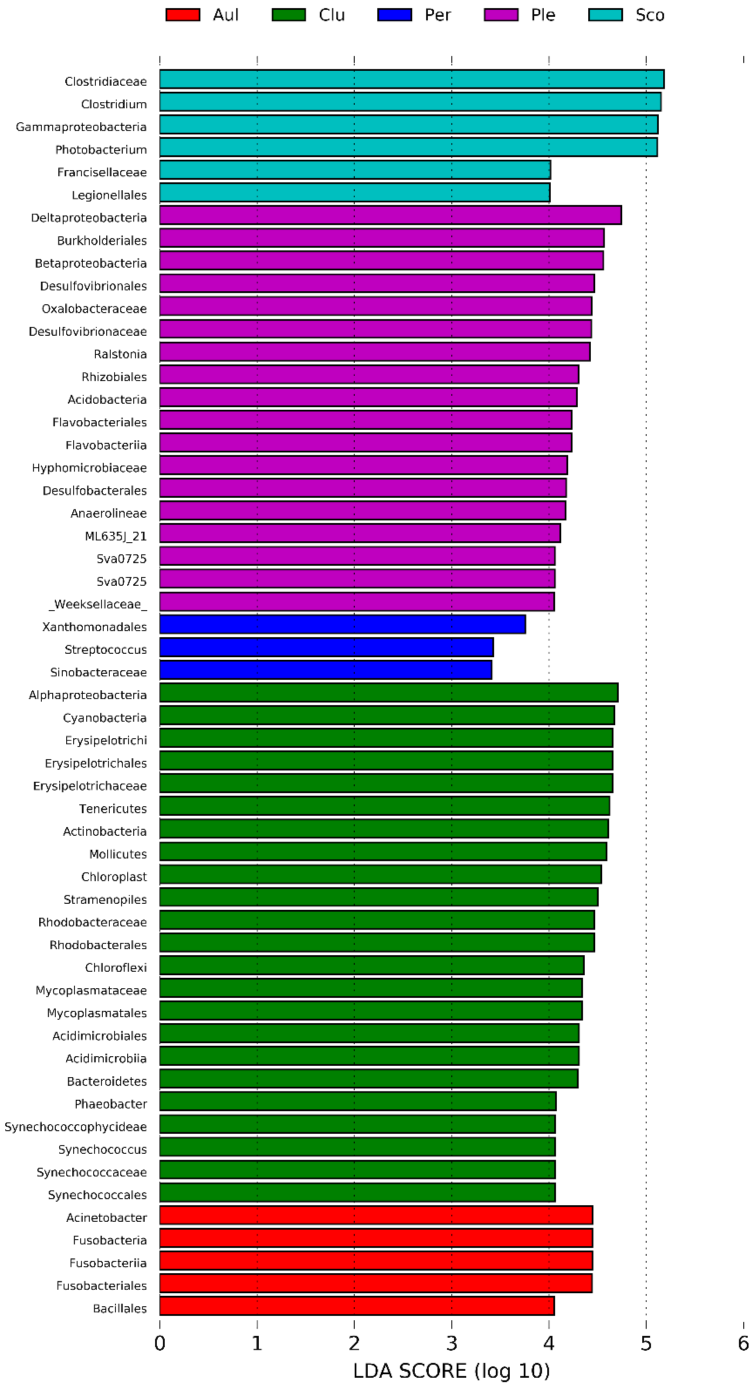

Fig. S5. Linear discriminant analysis (LDA) score of each discriminative bacterial clade identified with LEfSe.

MOLECULAR ECOLOGY

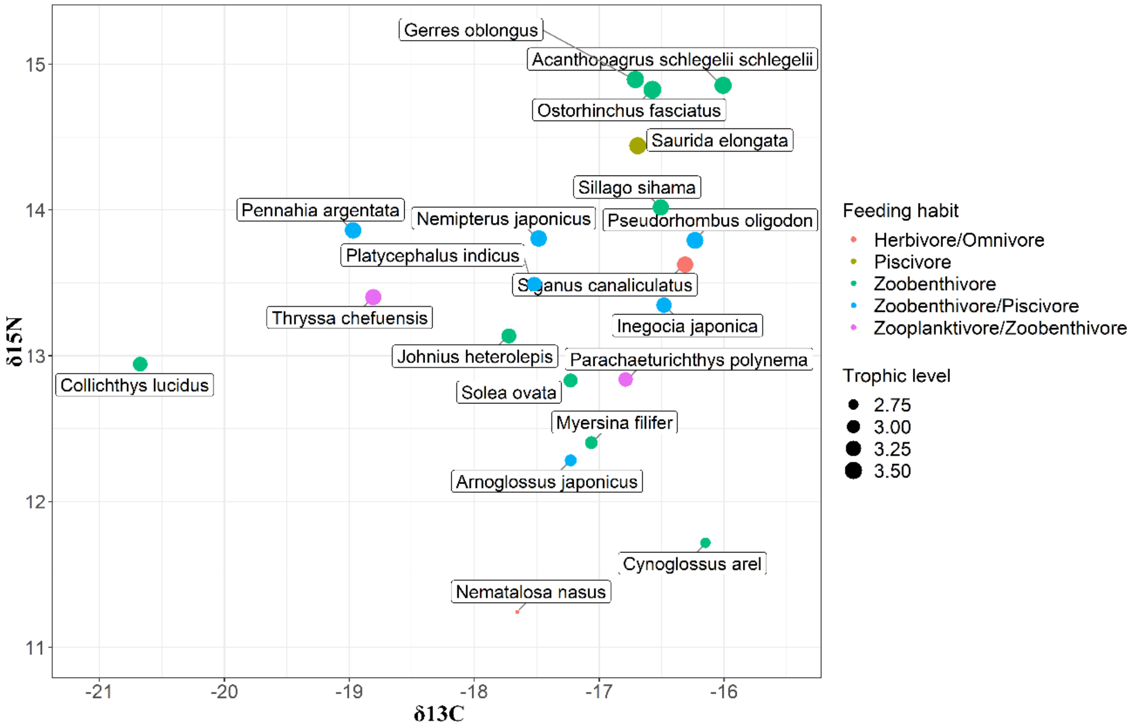

Fig. S6. Mean stable isotope C (δ^{13}C) and N (δ^{15}N) values identified in the dorsal muscle tissue of fishes. The calculated trophic levels are represented by the different shades of each color.

MOLECULAR ECOLOGY

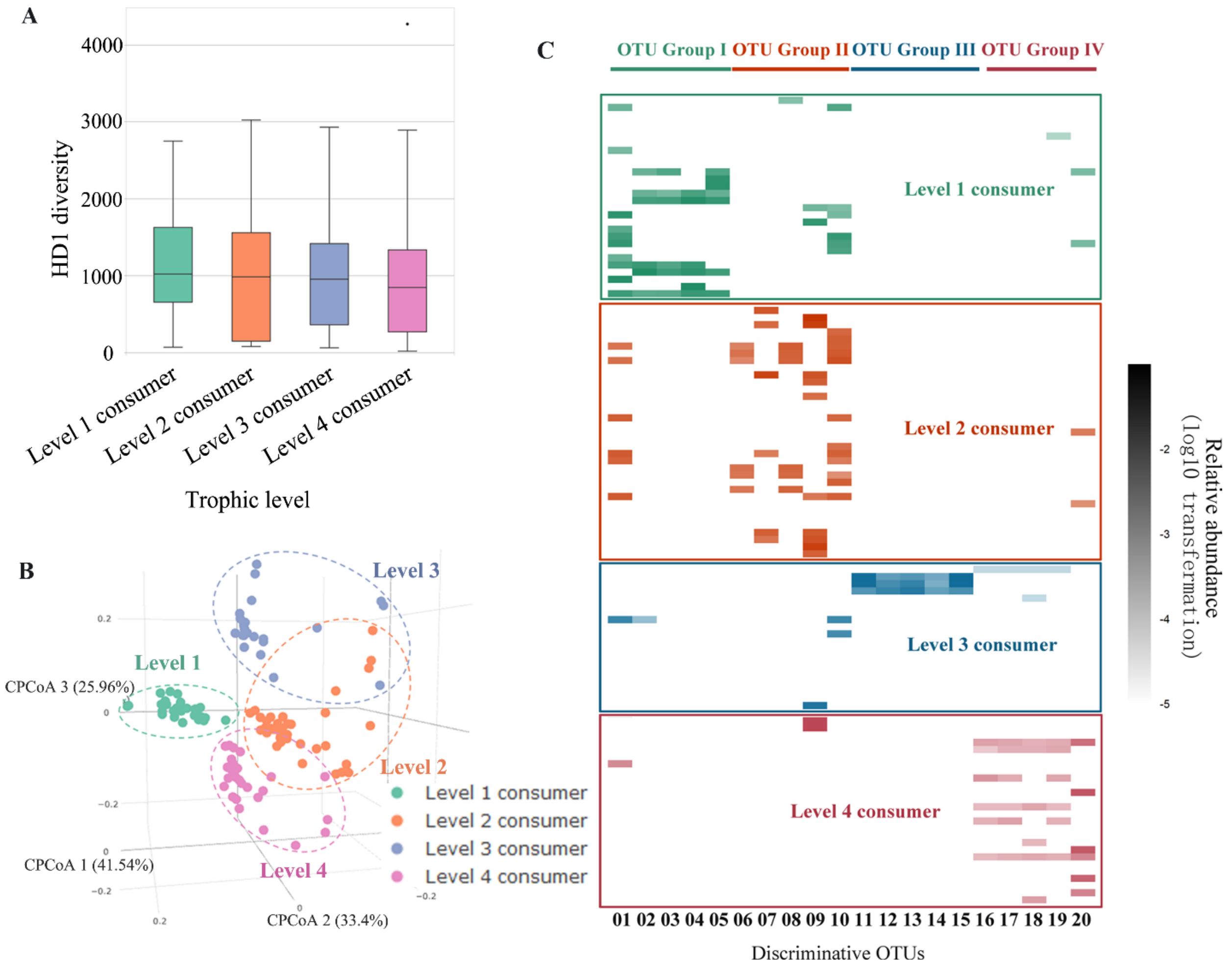

Fig. S7. The pattern of fish gut microbiomes shaped by trophic level. (A) Box plots of the alpha diversity of the different fish trophic level groups. (B) Clustering pattern of all fish gut microbiome samples categorized into 4 trophic level groups using canonical correlation analysis with Bray-Curtis distance. (C) Abundance heatmap of discriminative gut microbiomes identified among four distinguishing fish trophic level groups and the potential digestive functions (p-value < 0.01).

MOLECULAR ECOLOGY

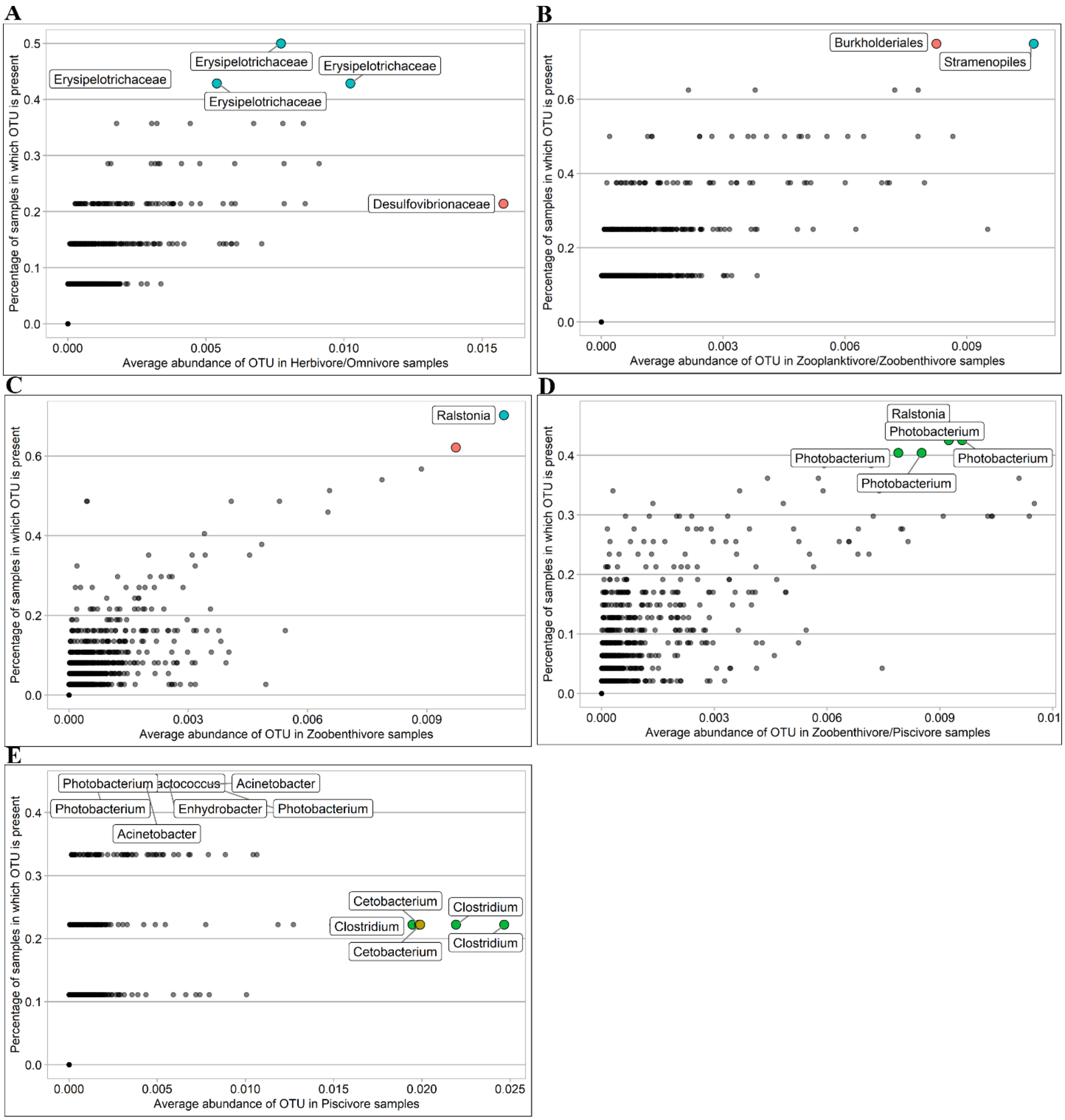

Figure S8. Abundance against prevalence of bacterial intra-feeding habits at the ASV level: (A) herbivore/omnivore, (B) zooplanktivore/zoobenthivore, (C) zoobenthivore, (D) zoobenthivore/piscivore, and (E) piscivore.

MOLECULAR ECOLOGY

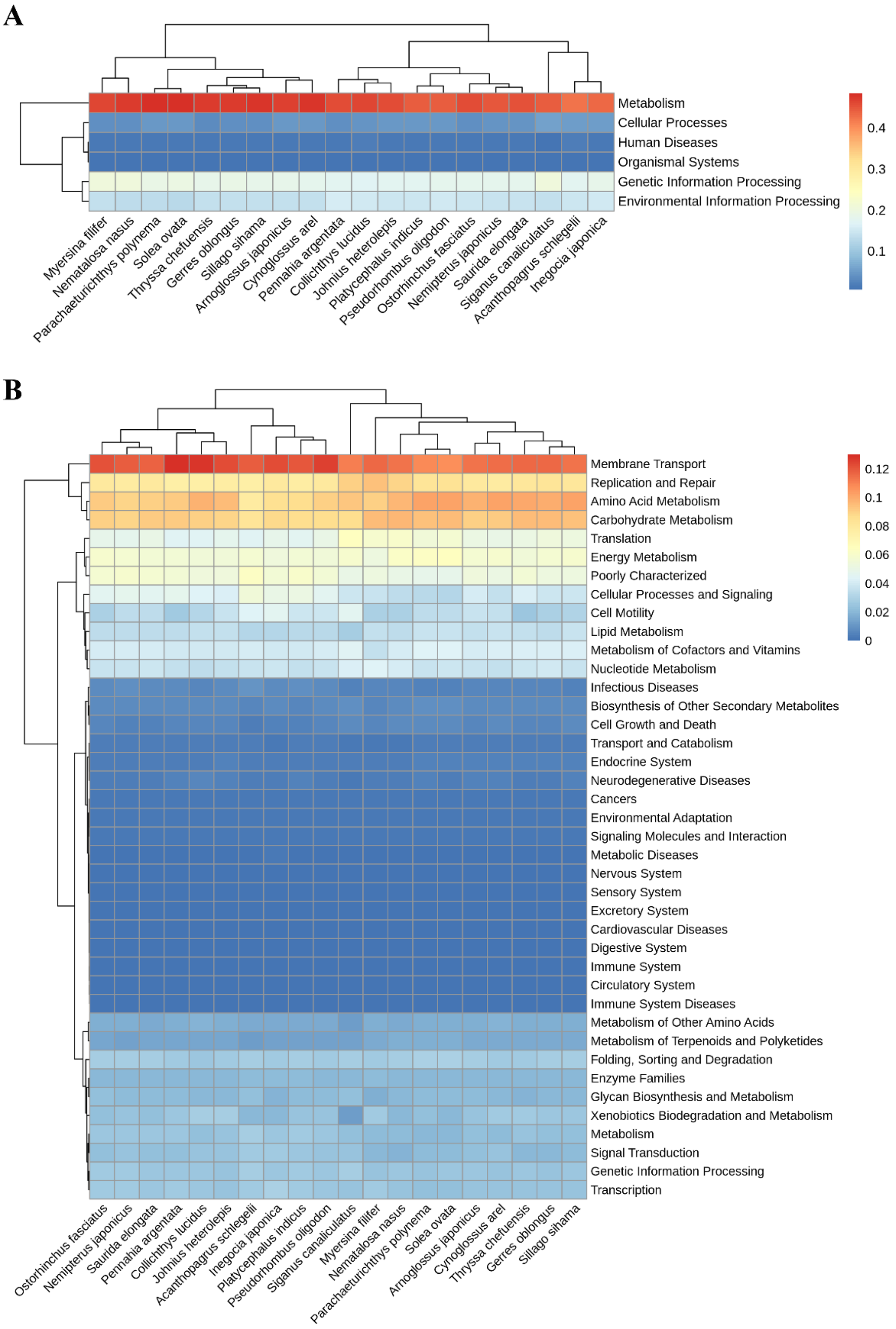

Figure S9. Heatmap of KEGG pathways. (A) Level 1 pathways. (B) Level 2 pathways.

MOLECULAR ECOLOGY

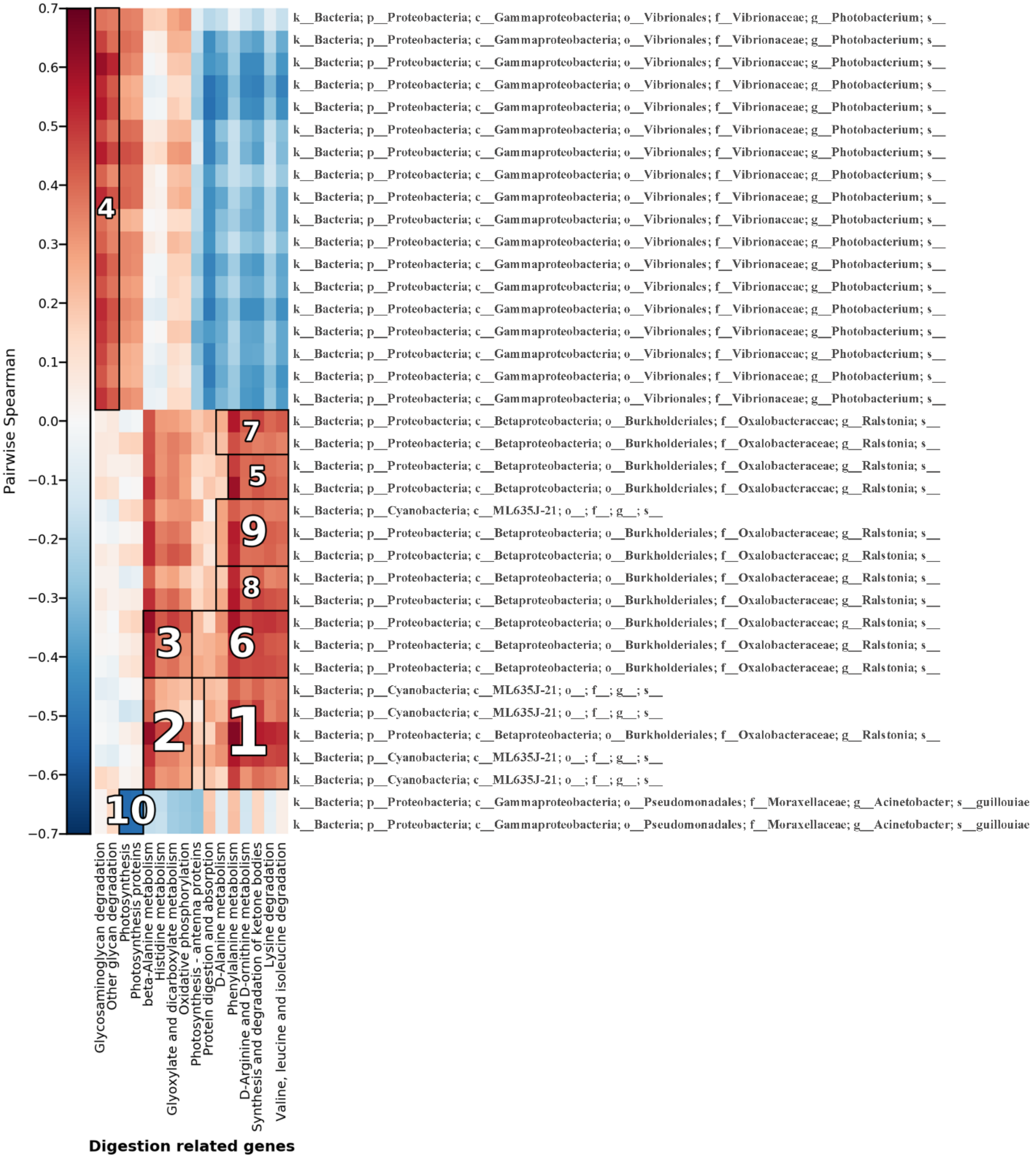

Figure S10. Association between fish gut microbiomes and digestion related genes.
