# Supplementary material for: Diversity of gut microbiomes in marine fishes is shaped by host‐related factors
Source: Mol Ecol. 2020 Nov 9;29(24):5019–34. doi: 10.1111/mec.15699 (PMC7756402; doi:10.1111/mec.15699)
Supplement: Supplementary file 3 — Appendix S1 [file MEC-29-5019-s003.pdf]

MOLECULAR ECOLOGY

Supplemental Information for:

Diversity of gut microbiomes in marine fishes is shaped by host-related factors

Qi Huang, Ronia C. T. Sham, Yu Deng, Yanping Mao, Chunxiao Wang, Tong Zhang, Kenneth M. Y. Leung

Table of Contents:

**Quality control during DNA extraction and PCR amplification**	Page 1-2

We supplemented two negative control samples to check the background and processing contamination. Negative control samples were useful in assessing, quantifying and removing background and processing contamination *in silico*. DNA extraction by FastDNA® SPIN Kit and full-length 16S rRNA gene PCR by primer 27F and 1492R were conducted. The DNA gel electrophoresis figure was shown below, which indicated no obvious full-length 16S rRNA gene band (~1500 bp) was observed in NC1 (negative control sample 1) and NC2 (negative control sample 2).

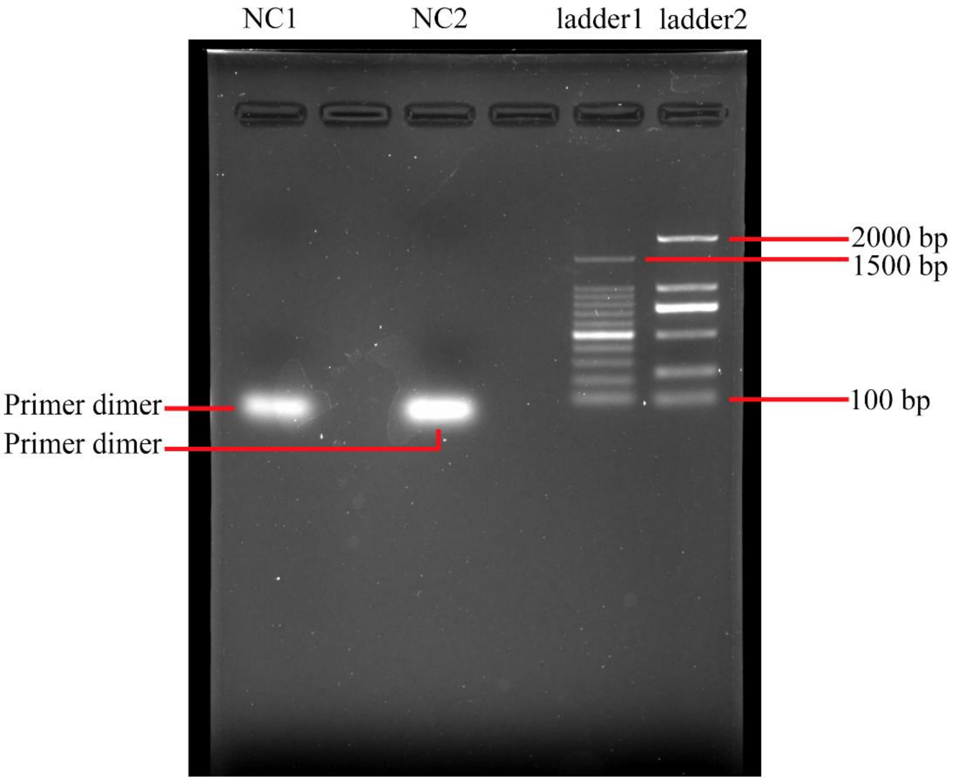

Additionally, considering that fish gut microbiomes were not low-biomass samples, there should be a less impact of the background contaminants on the extracted DNA. Despite that, we have utilized some strategies to physically minimize the contamination during DNA

extraction. We conducted the DNA extraction in a clean bench located in a physically separated room which was specifically used for DNA extraction and PCR. We sterilized the plastic consumables (i.e., pipette, pipette tips, Eppendorf) and the working bench with UV for 30 min before DNA extraction.

To avoid the well-to-well contaminant introduced during DNA extraction and PCR, several principles of the sample treatments suggested by Minich et al. (2019) were followed, i.e., manual single-tube extraction, consistent biomass and randomly distributed wells in PCR. First, we extracted DNA manually using individual Eppendorf tube rather than well-plate. The proportion of well-to-well contamination in manual single-tube extraction was generally lower than in plate-based extraction. Second, the biomass of gut microbiota samples from each species should be approximately consistent as they were the same sample type compared with other environmental samples such as water and sediment. Thus, the concern about higher well-to-well contamination in lower-biomass samples when extracted with higher-biomass samples could be relieved. Third, the 16S rRNA gene amplicon PCR runs were conducted following standardized methods with sound QA/QC by a commercial company which confirmed that they randomly distributed the samples in the well plate before the analysis.

Minich, J. J., Sanders, J. G., Amir, A., Humphrey, G., Gilbert, J. A., & Knight, R. (2019). Quantifying and Understanding Well-to-Well Contamination in Microbiome Research. *mSystems, 4*(4), e00186-00119. doi:10.1128/mSystems.00186-19
